# A randomized dietary intervention to increase colonic and peripheral blood SCFAs modulates the blood B- and T-cell compartments in healthy humans

**DOI:** 10.1093/ajcn/nqac246

**Published:** 2022-09-09

**Authors:** Paul A Gill, Jane G Muir, Peter R Gibson, Menno C van Zelm

**Affiliations:** Department of Gastroenterology and Department of Immunology and Pathology, Central Clinical School, Monash University and Alfred Hospital, Melbourne, Australia; Department of Immunology and Pathology, Central Clinical School, Monash University and Alfred Hospital, Melbourne, Australia; Department of Microbial Diseases, UCL Eastman Dental Institute, University College London, London, United Kingdom; Department of Gastroenterology and Department of Immunology and Pathology, Central Clinical School, Monash University and Alfred Hospital, Melbourne, Australia; Department of Gastroenterology and Department of Immunology and Pathology, Central Clinical School, Monash University and Alfred Hospital, Melbourne, Australia; Department of Immunology and Pathology, Central Clinical School, Monash University and Alfred Hospital, Melbourne, Australia

**Keywords:** dietary fiber, short-chain fatty acids, colonic fermentation, adaptive immunity, T cells, B cells, resistant starch

## Abstract

**Background:**

SCFAs have immune-modulating effects in animal models of disease. However, there is limited evidence that this may occur in humans.

**Objectives:**

This study aimed to determine the effects of increased exposure to SCFAs via dietary manipulation on colonic fermentation and adaptive immune cells.

**Methods:**

Twenty healthy young adults (18–45 y of age) underwent a blinded randomized crossover dietary intervention, consuming a high-SCFA diet and a matched low-SCFA diet for 21-d with a 21-d washout in between. SCFAs were provided through resistant starch, inulin, and apple cider vinegar. Blood and 3-d total fecal output were collected at baseline and at the end of each diet. GC was used to measure fecal and plasma SCFA. Flow cytometry was used for peripheral blood immunophenotyping.

**Results:**

According to a paired samples Wilcoxon test, the high-SCFA diet was associated with significantly higher fecal SCFA concentrations [median (IQR); 86.6 (59.0) compared with 75.4 (56.2) µmol/g, *P* = 0.02] and lower fecal ammonia concentrations [26.2 (14.7) compared with 33.4 (18.5) µmol/g, *P* = 0.04] than the low-SCFA diet. Plasma propionate [9.87 (12.3) compared with 4.72 (7.6) µmol/L, *P* = 0.049] and butyrate [2.85 (1.35) compared with 2.02 (1.29) µmol/L, *P* = 0.03] were significantly higher after the high-SCFA diet than after the low-SCFA diet. Blood total B cells [184 (112) compared with 199 (143) cells/µL, *P =* 0.04], naive B cells [83 (66) compared with 95 (89) cells/µL, *P* = 0.02], Th1 cells [22 (19) compared with 29 (16) cells/µL, *P* = 0.03], and mucosal-associated invariant T cells [62 (83) compared with 69 (114) cells/µL, *P* = 0.02] were significantly lower after the high-SCFA diet than the low-SCFA diet.

**Conclusions:**

Increasing colonic and peripheral blood SCFA has discrete effects on circulating immune cells in healthy humans following 3-wk intervention. Further studies (e.g., in patients with inflammatory disease) are necessary to determine whether *1*) these changes have immunomodulatory effects, *2*) they are therapeutically beneficial, and *3*) prolonged intake might be required. This trial is registered at the Australian New Zealand Clinical Trials Registry as ACTRN12618001054202.

## Introduction

SCFAs, predominantly acetate, propionate, and butyrate, are 2- to 4-carbon chain molecules that are primarily generated in the colon through fermentation of nondigestible dietary carbohydrates by gut microbiota (1). In addition, SCFAs are present in some foods and beverages via generation in fermentation processes that occur during their production (2). Increased generation of SCFAs in the colon and subsequent systemic delivery have been associated with improved colonic health and reduced metabolic disease and systemic inflammation, primarily in small animals (3). However, there have been difficulties translating these observations to therapeutic effects in humans, due to challenges with delivering adequate amounts of SCFA to the colon and inherent differences in SCFA metabolism and gut physiology between animals and humans (4).

Although SCFAs have potent effects on the immune system within animal models, evidence for the purported effects of SCFAs on the human immune system is primarily limited to studies in vitro, in which blood immune cells are exposed to SCFAs, often at concentrations well above physiologic levels (5). The few studies performed in vivo have been limited to using dietary fiber supplements, have lacked dietary controls, and have not observed changes to peripheral blood SCFA concentrations (6–8). Yet, dietary intervention studies that successfully used fiber supplementation to increase fecal and plasma SCFAs did not address effects on immune parameters (9, 10). Exposure to SCFAs may modulate the activity of the innate and adaptive immune responses through directly engaging SCFA-specific G protein–coupled receptors (GPR41, GPR43, GPR109a) on the cell surface or by entering the cell to alter cellular metabolism and histone deacetylase activity (11). Therefore, effects on a variety of immune cell lineages and subsets might be anticipated. We previously showed that a 5-d high-fiber diet that successfully increases plasma SCFA concentration did not change proportions of circulating regulatory T cells (Tregs) or concentrations of a panel of plasma cytokines (12). The increased exposure to SCFA in terms of concentrations achieved and/or its duration may have been insufficient to induce changes in healthy individuals. Altogether, there is a clear need for well-designed intervention studies to investigate if SCFA can indeed modulate the human immune system.

Fermentable fiber supplements, such as high-amylose maize starch and inulin, are an effective way to increase colonic SCFA production in human subjects and are amenable to use in a blinded study design since they have little taste themselves and take up flavors well (13). In addition, oral intake of fermented foods such as apple cider vinegar can be used to acutely deliver SCFAs to the systemic circulation (2). We hypothesized that supplementing a diet with a combination of dietary fermentable fiber [inulin and resistant starch (RS)] and oral SCFA (apple cider vinegar) would be a feasible strategy to increase colonic and plasma SCFA sufficiently to modulate aspects of the immune system in humans. Hence, the current study aimed to address this hypothesis with 3 major goals: first, to define the pharmacokinetics of blood SCFA concentrations during 3-wk consumption of a high-fiber diet with oral SCFA-rich foods; second, to assess changes to colonic fermentation resulting from increased fiber and oral SCFA intake; and third, to assess if this dietary intervention affects circulating immune cells in blood, particularly subsets of B and T lymphocytes shown to be modulated by SCFAs in preclinical models.

## Methods

### Participant recruitment

Healthy adults who were aged 18–45 y inclusive and believed themselves to be in good health were recruited for this study. Exclusion criteria included a history of gastrointestinal or other chronic inflammatory disease, recent acute illness or infection, antibiotic use within 1 mo of study, consumption of probiotics or prebiotics within 1 mo of study, use of medication that could alter gastrointestinal transit (e.g., laxatives or hypomotility agents), pregnancy or planning pregnancy, an eating disorder, special dietary requirements (vegetarian/vegan), or current use of medication for hypertension. Volunteers who expressed interest in the study were screened with an eligibility questionnaire, followed by a face-to-face meeting with the study coordinator to obtain written informed consent.

### Study design

**FIGURE 1:**
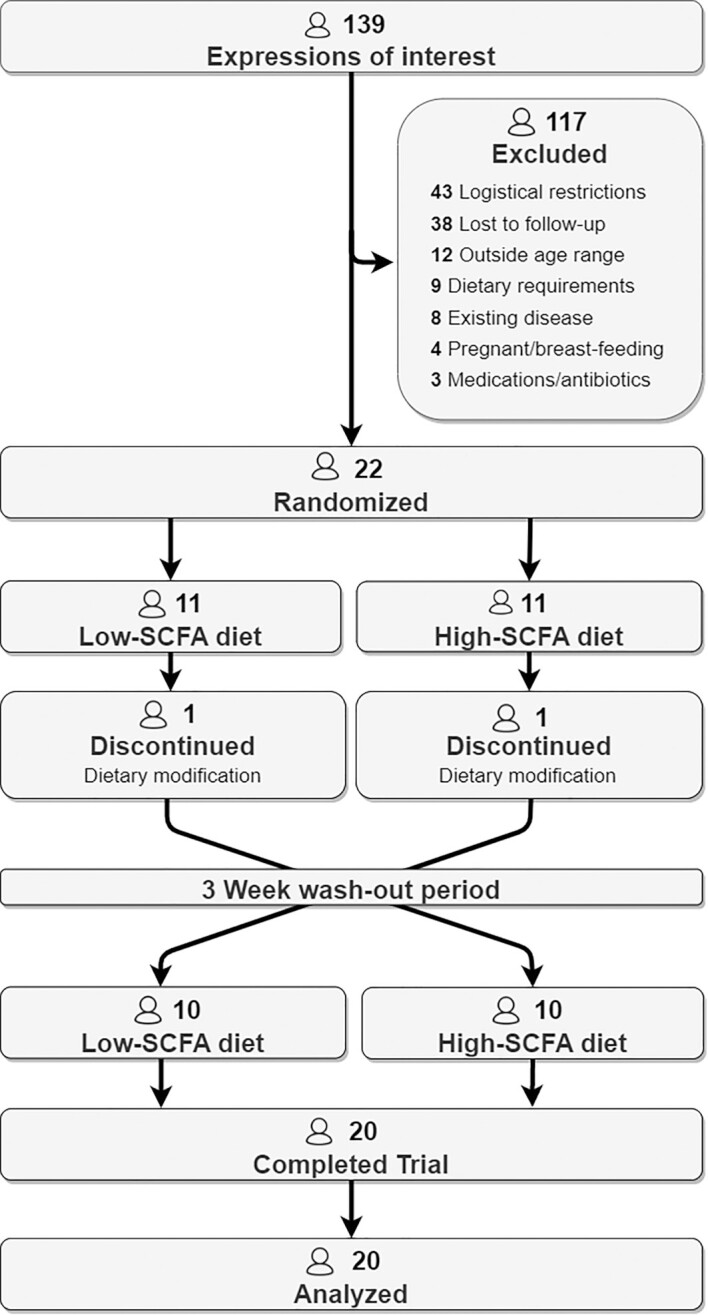
Participant flow diagram for intervention study. Of 139 expressions of interest, 22 healthy participants were randomly assigned to intervention. A total of 20 participants completed the study, with 2 dropouts after the first arm.

The study was designed as a dietary intervention composed of a randomized single-blinded crossover trial, as outlined in **Figure 1**. Each study participant undertook a 1-wk baseline (BL) period before being assigned via a random number generator (version 4; http://www.randomizer.org/) to either a low-SCFA (L-SCFA) or high-SCFA (H-SCFA) diet for 3 wk. Afterward, participants underwent a minimum 3-wk washout period where they were free to consume their habitual diets prior to completing the other 3-wk interventional diet. Subjects but not the investigators were blinded to the nature of the diet. On the morning of day 1 of the BL week, participants had 30 mL of peripheral venous blood collected. They also undertook 3-d total fecal collection during the BL week and completed a food and gastrointestinal symptom diary. This diary was also completed throughout the 3-wk dietary intervention period. At the end of the dietary intervention period, participants again had blood collected on the morning of day 20 or 21. They also completed 3-d total fecal collection on days 18–20 of the intervention period. Weight measurements were recorded with a digital physician scale (Tanita) during each study visit at Alfred Hospital, Melbourne, Australia. The study protocol was approved by the Monash University Human Research Committee (project no. 11190) and prospectively listed on the Australian New Zealand Clinical Trials Registry (ACTRN12618001054202).

### Intervention diets

H-SCFA and L-SCFA diets were developed to meet the standards of the Australian Dietary guidelines (14) and provided equivalent levels of carbohydrates, proteins, fats, and other micronutrients. Nutritional composition was assessed using Foodworks Professional software (version 7.01; Xyris) with data from the NUTTAB (Nutrient Tables for Use in Australia; Food Standards Australia New Zealand) and Monash University FODMAP databases. The meal plans for both diets are shown in **Table 1**. The L-SCFA diet provided 18.6 g fiber/d, of which 1 g/d was RS and 1 g/d was nondigestible oligosaccharide. The H-SCFA diet contained an additional 20 g high-amylose maize starch/d (HAMS-1043; Ingredion) that was added to 2 food items, as indicated in Table 1. This was equivalent to approximately 10 g RS/d (15). In addition, 8 g of inulin [average degree of polymerization ≥10 (OraftiGR; Beneo)] was included in a daily meal. Taken together, the H-SCFA diet provided an additional 18 g of fermentable fiber each day. The macronutrient and fiber breakdown of the H-SCFA and L-SCFA diets is presented in **Table 2**. To boost SCFA delivery, an apple cider vinegar drink consisting of apple cider vinegar (Melrose) mixed with an equal volume of flavored syrup (Cottee's Blackcurrant Cordial) was provided to participants to consume after each main meal (breakfast, lunch, dinner). Each drink contained approximately 25.8-mmol acetate, 0.05-mmol propionate, and 0.04-mmol butyrate as previously described (2). A placebo vinegar drink was provided on the L-SCFA diet that contained flavored syrup and approximately 0.01% citric acid (McKenzie's). This was pH matched to the H-SCFA vinegar drink. Participants consumed 40 mL of this vinegar drink with 250 mL of water to prevent irritation of the esophagus. All meals were prepared, cooked, packed, and frozen under the supervision of qualified chefs in a licensed commercial kitchen with storage facilities [BASE Facility (Be Active Sleep Eat), Monash University]. Study food was packed in freezer bags and delivered to participants, who recorded their intake into a food diary as a measure of adherence to the diet. The study researcher provided a list of additional snacks (**Supplemental Table 1**) that could be consumed to improve adherence to each diet. Furthermore, the study coordinator contacted study participants either in person or by phone each week to maintain adherence to the study diet.

**TABLE 1:** Meal plan of dietary intervention study^1^

Meal ^2^	Day 1	Day 2	Day 3	Day 4	Day 5	Day 6	Day 7
Breakfast	**Breakfast smoothie with oats (RS)** and berries; kiwifruit	**Pancake (RS)** with berries and yogurt	White bread toast with spread; tub of yogurt	**Breakfast muffin (RS)** with eggs and tomato	**Pancake (RS)** with berries and yogurt	**Breakfast smoothie with oats (RS + I)** and berries; kiwifruit	**Breakfast muffin (RS)** with eggs and tomato
Morning tea	Sweet biscuits	1 medium kiwi fruit, 2 sweet biscuits	**Savory muffin (RS)**	Peach and peanuts	Sweet biscuits	Almonds	Sweet biscuits
Lunch	Corn tortilla wrap (**meatballs**, spread, cheese, salad)	**Arancini (RS)** and salad	**Pumpkin soup (I)** with white bread	White bread sandwich with cold meat, cheese, salad	**Beef curry (I)** with white rice	**Tuna sweet potato patties (RS)** and salad	White bread sandwich with cold meat, cheese, salad
Afternoon tea	**Sweet muffin (RS)**	Peanuts	Almonds	**Sweet muffin (RS)**	**Savory muffin (RS)**	Mandarin and sweet biscuits	**Smoothie with oats (RS)** and berries
Dinner	**Chicken cacciatore (I)** with white rice	**Bolognaise (I) with pasta** and salad	**Frittata (RS)** with grilled meat and salad	**Chicken risotto (I)**	**Vegetable stir-fry** with rice noodles	**Rice pilaf** with grilled meat	**Pasta with tomato and bacon sauce (I)**

1 Bold indicates that the meal was provided. I, inulin; RS, resistant starch.

2 Vinegar or placebo drink was consumed after breakfast, lunch, and dinner.

**TABLE 2:** Nutrient breakdown of intervention diets

	Daily intake	
Component	Low-SCFA	High-SCFA
Energy, kJ	8687	8660
Carbohydrate, g	212.7	216.0
Sugars, g	68.4	69.0
Protein, g	98.2	98.0
Fat, g	84.2	84.4
Saturated fat, g	29.0	29.0
Sodium, mg	3391	3397
Total dietary fiber,^1^ g	18.6	39.2
Resistant starch, g	1.0	11.0
Inulin, g	0	8.0
Oligosaccharides,^2^ g	1.0	1.0
Energy intake, % total energy intake		
Carbohydrate	41.6	42.4
Fat	35.9	36.0
Protein	19.2	19.2

1 Total dietary fiber includes resistant starch, inulin, and oligosaccharides in the supplied background diet.

2 Oligosaccharides: fructans + galacto-oligosaccharides.

### Dietary and symptom analysis

Participants completed 7-d food diaries at BL, whereby they recorded all food and drinks consumed. Scored measuring cups and spoons were provided to participants to assist with this process. In addition, to improve the accuracy of data collection, participants recorded information on brands of food items consumed and documented recipes of meals cooked. Information contained in these diaries was first analyzed with Foodworks Professional (version 9.0) to calculate energy, macronutrient, and micronutrient intake and then analyzed with additional data from the Monash University FODMAP database (which includes oligosaccharide information) and an RS composition report (16) to generate detailed information on fiber intake.

During the BL week and intervention diets, participants scored daily overall abdominal symptoms, abdominal pain, bloating, passage of wind (gas), satisfaction with stool consistency, tiredness, and nausea using 100-mm visual analog scales, as previously applied (17). Participants also recorded the number of bowel movements they had each day and their stool form based on the Bristol Stool Scale (BSS).

Compliance to the intervention diet was assessed by examining the fold differences in RS and inulin intake from the L-SCFA to H-SCFA diets relative to the 18-fold change designed. Participants who achieved 80% of this expected fold change (equivalent to 15.2-fold change) were considered compliant to the intervention diets.

### Fecal collection and analysis

Participants collected all fecal output for 3 d by placing freshly passed stools into provided plastic containers. These were sealed with a lid, sealed in another bag, and immediately placed into a portable freezer set at –20ºC. After the collection period, stool samples were transported to the laboratory at –20ºC for storage and processing. All fecal material collected was weighed to obtain 3-d and daily fecal output. The samples were thawed, pooled, and homogenized. Aliquots were subsequently stored at –80ºC. Fecal water content was estimated as previously described (18) and expressed as a percentage of the initial wet weight of sample.

Fecal pH was measured with a calibrated pH probe (Mettler Toledo) inserted into thawed fecal material warmed to 25ºC. Fecal ammonia was measured in duplicate via an enzymatic assay (Megazyme rapid ammonia assay) after protein precipitation and filtration as previously described (17). Fecal SCFAs were measured in triplicate via GC as previously described (19). Thawed fecal material was spiked with 3 times the volume of internal standard (1.68mM heptanoic acid), homogenized and centrifuged (2000 × *g*, 10 min, 4°C), after which 300 µL of supernatant was added to a 0.2-µm filter vial containing 10 µL of 1M phosphoric acid. The vials were then analyzed for SCFA content via GC using an Agilent GC6890 coupled to a flame ionization detector. CV <10% was taken as a valid result for fecal ammonia and SCFA concentrations (micromoles per gram). Daily fecal output was used to calculate daily excretion of SCFAs (millimoles per day).

### Blood collection

Peripheral blood samples were collected in EDTA-coated vacutainers. Within 4 h of collection, plasma was collected, and peripheral blood mononuclear cells (PBMCs) were isolated with a Ficoll-Paque gradient. PMBCs were cryopreserved in liquid nitrogen, whereas aliquots of plasma were stored at –80ºC for later use. A 50-µL volume of peripheral blood was used to perform immune cell counts (see Flow Cytometry section).

### Plasma SCFA analysis

Blood plasma was analyzed in triplicate for SCFA content using GC as previously described (12). Briefly, 300 µL of plasma was spiked with 200µM heptanoic acid and acidified with 10% sulfosalicylic acid before the addition of diethyl ether solvent. The mixture was vortexed and centrifuged so that the organic layer could be clarified and transferred to 0.2M NaOH. The alkaline solution containing SCFA was concentrated by evaporation via nitrogen, dissolved in 1M phosphoric acid, and transferred into a cold GC glass vial for analysis using an Agilent GC6890 coupled to a flame ionization detector. Concentrations for acetate, propionate, and butyrate were determined by the average of the triplicate results, where the CV was <20%. Total SCFA was calculated by the sum of the individual SCFAs. Results were expressed as micromoles per liter. Data points below the limit of detection were excluded from relative change calculations.

### Flow cytometry

Absolute numbers of major leukocyte subsets (granulocytes, monocytes, B cells, T cells, and NK cells) were determined with a lyse-no-wash method within 24 h of blood sampling as previously described (20). In brief, 50 µL of whole blood was added to a TruCount tube (BD Biosciences) with 20 µL of antibody cocktail (CD3FITC, CD45-PerCP-Cy5.5, CD16-PE, CD56-PE, CD4-PE-Cy7, CD19-APC, APC-H7) for 15 min in darkness (gating strategy in **Supplemental Figure 1**). Red blood cells were lysed with the addition of 500 µL of ammonium chloride solution (0.155 M) prior to analysis on an LSRII flow cytometer (BD Biosciences). A small aliquot of whole blood was also analyzed using a Cell-Dyn Emerald instrument (Abbott) to cross-reference leukocyte numbers enumerated with flow cytometry.

PBMCs collected during the study were thawed and subjected to 2 staining panels to evaluate B-cell (21) and T-cell lineages and subsets therein. Stored PBMCs were thawed from liquid nitrogen storage in a water bath at 37ºC and resuspended in RPMI containing 10% fetal calf serum. Viable PBMCs were enumerated with a Cellometer K2 instrument (Nexcelom Bioscience) and resuspended in FACS wash buffer at a concentration of 4.0 × 10^7^ cells/mL prior to staining, with markers outlined in **Supplemental Table 2**. For the B-cell tube, 2.0 × 10^6^ cells were stained, and 1.0 × 10^6^ cells were included in the T-cell tube. Stained cells were run on a 5-laser LSR Fortessa X-20 flow cytometer (BD Biosciences). Instrument setup and calibration were performed as previously described (20), with additional optimization for the BUV395, BUV496, BUV737, and BV785 channels. All data were analyzed via Flow-Jo software (version 10.6; BD Bioscience).

### Statistical analysis

A sample size calculation was initially performed according to preliminary data (Treg as a percentage of CD4^+^ T cells) collected during a pilot study (12) to estimate the number of participants needed to observe a statistically significant difference in Treg number (20% change, effect size = 0.55) between the H-SCFA and L-SCFA dietary interventions (G*Power version 3.1). The minimum sample size required was 30 participants for a statistical power equal to 0.8 and an α level of 0.05 (2-tailed). With a 10% dropout rate, the sample size required was calculated as 33 participants.

Statistical analysis was performed using Prism (version 8.02; GraphPad) and R (version 3.6.1) on a per-protocol basis. The primary endpoint examined changes to Treg number. Secondary endpoints included assessment of fecal metabolites and characteristics, plasma SCFA concentration, and other blood immune cell subsets. Endpoints were compared between the L-SCFA and H-SCFA diets using the paired samples Wilcoxon test. Correlation analysis between variables was calculated with the Pearson correlation test, with a correlation matrix assembled for all parameters recorded. False discovery rate corrections were applied to correlation data via the Benjamini and Hochberg method to correct for multiple comparisons. Immune network analysis was conducted with the R package Rtnse (version 0.15; https://github.com/jkrijthe/Rtsne), with perplexity set to 10. The significance level for this study was determined at 0.05.

## Results

### Study cohort

Of the 126 individuals who expressed interest in the study, 22 met the criteria. Major reasons for ineligibility were logistical restrictions (*n* = 43), being outside the age range (*n* = 12), dietary requirements (*n* = 9), and preexisting disease (*n* = 8); thus, the original sample size of 30 participants was not reached. Of the 22 participants who were randomly assigned, 2 withdrew after completing 1 arm, both due to additional dietary requirements that arose during the washout period. A total of 20 participants completed the study between August 2018 and September 2019, as illustrated in Figure 1.

**Table 3** contains the BL demographic, anthropometric, and habitual dietary characteristics of the 20 participants who completed the protocol according to sex. They were predominantly young; 12 were female; most were of normal weight; and all were normotensive. Median (IQR) energy intake of the study cohort was 9345 (2396) kJ/d, and total dietary fiber intake was 25.2 (12.3) g/d, this being in the range recommended by the Australian dietary guidelines (25–30 g/d).

**TABLE 3:** Baseline characteristics of study participants who completed the protocol according to sex^1^

	Male (*n* = 8)	Female (*n* = 12)
Age, y	30 (25–36)	25 (18–43)
Body mass index, kg/m^2^	24 (20.2–26.2)	22.0 (17.3–31.9)
Habitual dietary intake per day		
Energy, kJ	9949 (9143–12,343)	8252 (6377–11,831)
Carbohydrate, g	269 (233–377)	230 (52–327)
Sugars, g	111.3 (56.2–173.0)	67.5 (26.6–131.5)
Protein, g	114.1 (103.1–128.0)	87.7 (59.6–111.3)
Fat, g	89.5 (65.8–100.7)	72.1 (45.1–140.0)
Saturated fat, g	31.3 (20.0–47.2)	30.4 (13.6–64.6)
Sodium, mg	2063 (1176–3058)	2141 (1230–3639)
Total dietary fiber,^2^ g	28.5 (17.1–45.1)	23.6 (9.2–31.8)
Resistant starch, g	3.5 (3.2–7.9)	2.1 (1.2–4.4)
Oligosaccharides, g	4.0 (2.3–7.8)	3.2 (1.4–4.7)
Proportion of total energy intake, %		
Carbohydrate	46.4 (41.4–53.0)	43.3 (37.6–55.3)
Fat	30.0 (26.6–39.0)	34.4 (20.4–43.8)
Protein	18.8 (15.9–22.1)	17.2 (13.5–22.5)

1 Data are shown as median (range).

2 Total dietary fiber includes resistant starch and oligosaccharides.

### Dietary intake and adherence

**FIGURE 2:**
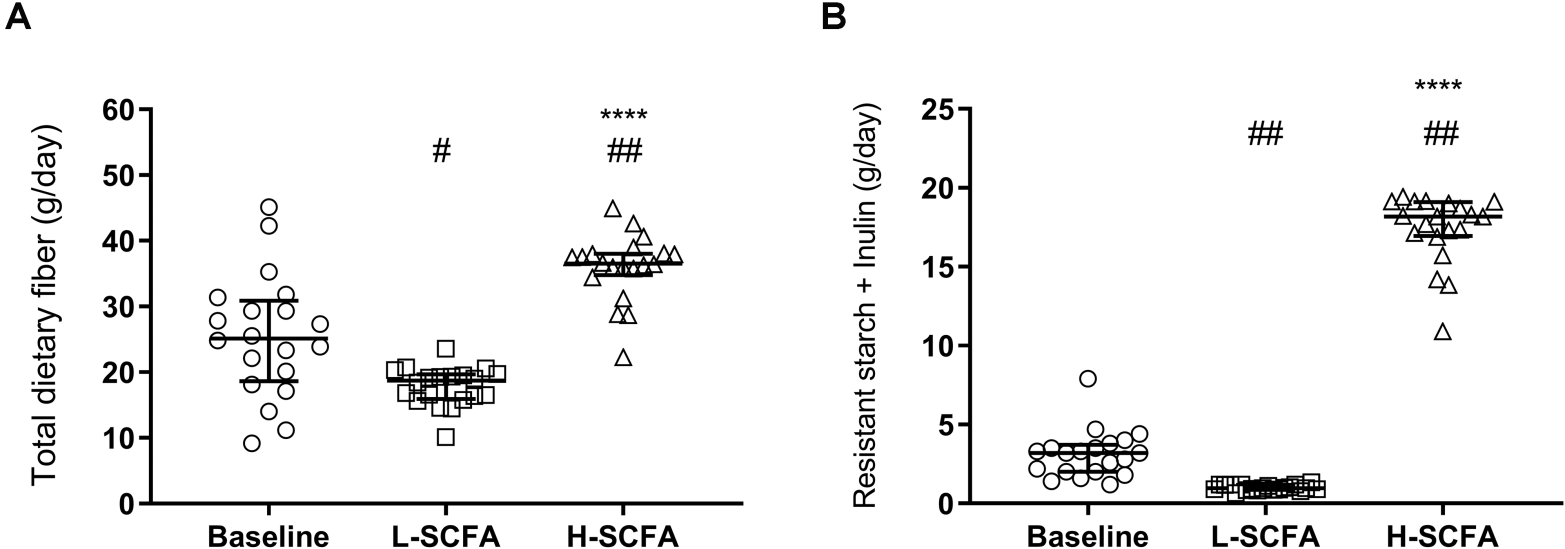
Dietary fiber intake during study intervention periods. Daily intake of (A) total dietary fiber and (B) resistant starch and inulin at baseline and during the intervention diets: low-SCFA (L-SCFA) and high-SCFA (H-SCFA). Data are shown as median (IQR). ^****^Different from low-SCFA, *P* < 0.001 (paired samples Wilcoxon test). Different from baseline: ^#^*P* < 0.05 and ^##^*P* < 0.01 (Friedman test). *N* = 20 matched pairs.

Dietary consumption of the study diets is shown in **Figure 2**. Median (IQR) overall fiber intake was 29.5 (3.3) g/d with the H-SCFA diet, which was greater than the 18.7 (3.8) g/d with the L-SCFA diet (*P <* 0.001). This was primarily due to a greater intake of RS [10.5 (1.1) compared with 1.0 (0.3) g/d, *P* < 0.0001] and inulin [7.4 (1.0) compared with 0 (0) g/d, *P* < 0.0001] with the H-SCFA diet. Daily macronutrient intake (grams per day) was similar between the H-SCFA and L-SCFA diets (**Supplemental Table 3**). Overall median dietary fiber and RS intake was also significantly greater during the H-SCFA diet and significantly lower during the L-SCFA diet when compared with the habitual diet consumed during the BL period (Figure 2B). The weight of the participants was stable across the study periods (data not shown).

Of the 20 participants, 18 were adherent and consumed >80% of the expected fold change increase in RS and inulin intake between the intervention diets (**Supplemental Figure 2**). Two participants achieved less than the minimum 80% cutoff with a 14.3- and 14.6-fold change increase, respectively. Similarly, adherence to the prescribed 3 vinegar drinks per day was high. Thus, during the L-SCFA diet, 18 of 20 participants reported consuming the allocated 3 drinks each day, with the remaining 2 participants consuming on average 2 drinks each day. During the H-SCFA diet, 16 of 20 participants consumed 3 drinks each day, with the remaining 4 consuming 2 drinks each day. When blinding was formally assessed upon completion of the study, 75% (15/20) of participants correctly recognized each diet. The vinegar drink containing apple cider vinegar was also correctly chosen by 80% (16/20) of the study cohort.

### Effects of intervention on gastrointestinal symptoms and fecal characteristics

The study diets were well tolerated, with overall symptom scores generally low throughout the intervention periods. There were no significant changes in overall or individual self-reported symptoms on the visual analog scale from BL to intervention diets except for a greater but small-magnitude difference in passage of wind/gas between the H-SCFA diet [median (IQR), 11 (12) mm] and the L-SCFA diet [7 (12) mm, *P* < 0.05; **Supplemental Figure 3**]. Upon formal assessment at the conclusion of the study, 74% of the cohort identified the H-SCFA diet as causing more abdominal symptoms.

The fecal characteristics are shown in (**Table 4**). The frequency of bowel movements and self-reported BSS scores were similar between the diets, although the proportion of stools with a BSS score of 3–5 during the H-SCFA diet was marginally higher than during the L-SCFA diet (*P* < 0.05). No differences were observed between the diets in 3-d or daily fecal output, fecal water content, and fecal pH.

**TABLE 4:** Participant bowel habit and fecal characteristics during study intervention periods^1^

Parameter	Low-SCFA	High-SCFA	*P* Value
Bowel movements/d	1 (1–3)	1 (1–2)	0.99
BSS score	3 (2–5)	4 (1–5)	0.84
Score 3–5, %	79 (0–100)	83 (5.3–94.7)	0.01^2^
Fecal^3^			
Total output, g	274 (107–755)	335 (58–743)	0.35
Output, g/d	91 (36–252)	112 (19–248)	0.35
Water content, %	72 (57–80)	71 (62–84)	0.10
pH	6.7 (6.3–7.5)	6.7 (6.1–7.5)	0.44

1 Data are shown as median (range). *N* = 20 matched pairs, unless indicated otherwise. BSS, Bristol Stool Scale.

2 Paired samples Wilcoxon test.

3 *n* = 19 matched pairs.

### Effect of interventions on fecal metabolites

**FIGURE 3:**
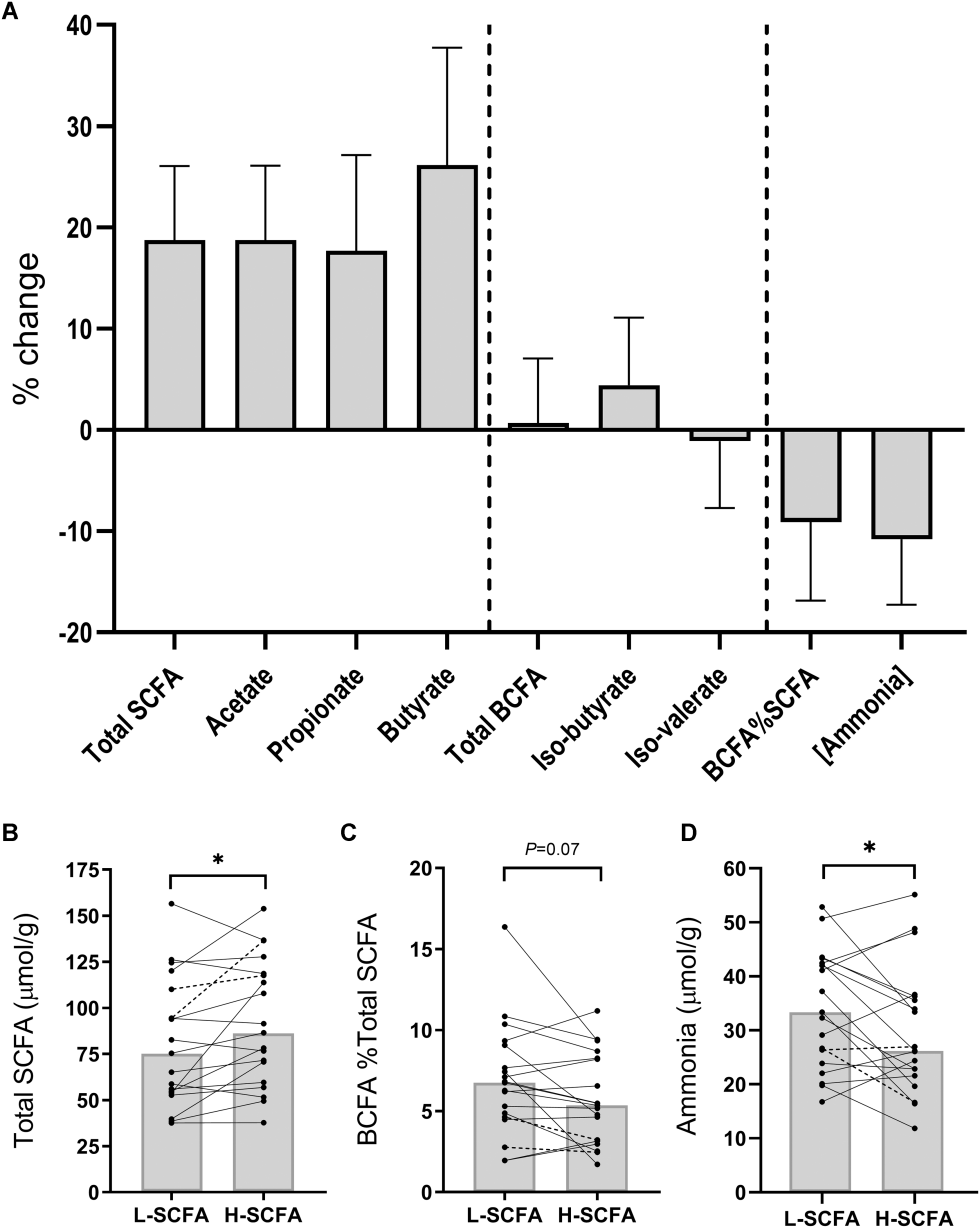
Fecal metabolite changes during intervention diets. (A) Percentage changes [(H-SCFA – L-SCFA)/L-SCFA] to fecal SCFA, BCFA, and ammonia as measured by GC. Data are shown as mean ± SEM. (B) Paired total fecal SCFA concentrations. (C) Paired ratio of amounts of BCFA to SCFA. (D) Paired fecal ammonia concentration as measured via enzymatic assay. Dotted lines represent participants with reduced compliance to study diet. Bar represents median. *P < 0.05 (paired samples Wilcoxon test). *n* = 19 matched pairs. BCFA, branched-chain fatty acid; H-SCFA: high-SCFA; L-SCFA, low-SCFA.

Fecal concentrations of SCFAs are shown in **Supplemental Table 4**. Total SCFA concentrations were 19% ± 7.3% higher (mean ± SEM percentage change) with the H-SCFA diet as compared with the L-SCFA diet as reflected in the acetate and butyrate concentrations (**Figure 3**A). This was in part due to a significantly higher median (IQR) acetate concentration [48.8 (39.9) compared with 41.4 (34.9) µmol/g, *P* < 0.05) and a trend toward a higher butyrate concentration. Overall total SCFA concentration was significantly higher after the H-SCFA diet [86.6 (59.0) µmol/g] than after the L-SCFA diet [75.4 (56.2) µmol/g; Figure 3B]. However, the ratio of butyrate to total SCFAs was similar between the L-SCFA diet [14.9% (4.3%)] and H-SCFA diet [15.2% (6.4%); data not shown]. Furthermore, the ratio of the total amount of branched-chain fatty acid to SCFA (Figure 3C) produced tended to be lower (*P* = 0.07) after the H-SCFA diet [5.4% (5.1%)] than after the L-SCFA diet [6.8% (4.5%)]. The fecal ammonia concentration was 33.4 (18.5) µmol/g after the L-SCFA diet, which was significantly higher than the 26.2 (14.7) µmol/g after the H-SCFA diet (*P* < 0.05; Figure 3D).

### Effect of interventions on plasma SCFA

**FIGURE 4:**
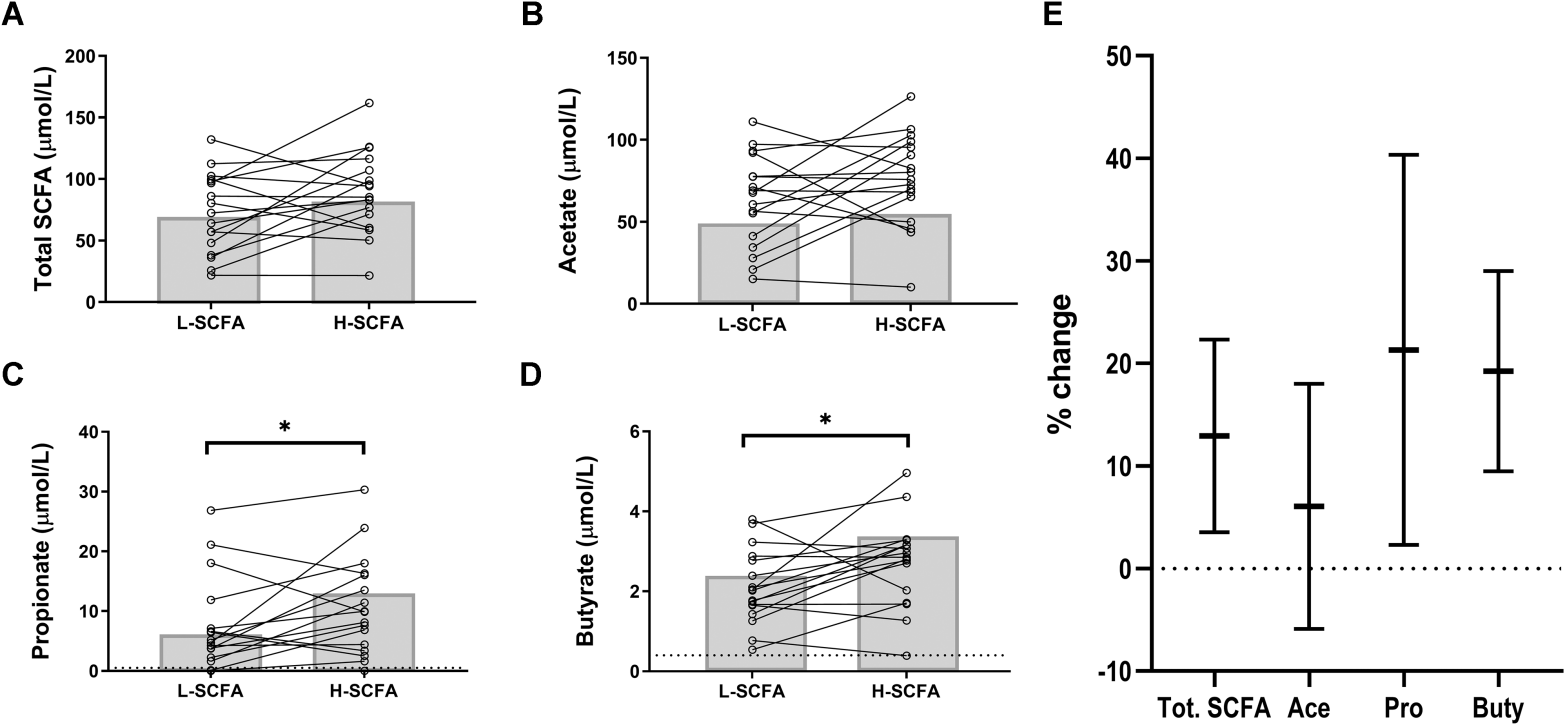
Plasma SCFA concentration after intervention diets. Paired concentrations of plasma (A) total SCFA, (B) acetate, (C) propionate, and (D) butyrate as measured by GC after intervention diets. Dotted line represents limit of detection for propionate and butyrate. Bar represents median. (E) Percentage changes to plasma SCFAs [(H-SCFA – L-SCFA)/H-SCFA]. Data are shown as mean ± SEM. Data points below the limit of detection excluded from percentage change calculations. Statistical significance calculated by paired samples Wilcoxon test. *n* = 17 matched pairs. H-SCFA, high-SCFA; L-SCFA, low-SCFA.

There were no significant differences in plasma total SCFA (*P =* 0.11) and plasma acetate (*P* = 0.22) between the intervention diets (**Figure 4**A, B). However, median (IQR) plasma propionate [9.87 (12.3) compared with 4.72 (7.6) µmol/L] and butyrate [2.85 (1.35) compared with 2.02 (1.29) µmol/L] concentrations were significantly higher after the H-SCFA diet than after the L-SCFA diet (Figure 4C, D). Furthermore, percentage change in plasma propionate and butyrate was a mean ± SEM increase of 21% ± 19% and 19% ± 9%, respectively, with the H-SCFA diet as compared with L-SCFA diet (Figure 4E). The proportions of plasma acetate, propionate, and butyrate within total SCFA were similar after the intervention diets (data not shown).

### Effect of interventions on circulating immune cells

Following the 3-wk H-SCFA and L-SCFA diet interventions, no differences were observed in total Treg number [median (IQR), 44 (29) compared with 45 (22) cells/µL, *P =* 0.25; **Supplemental Figure 4**] or Treg frequency within T cells [3.5% (1.2%) compared with 3.8% (1.5%), *P* = 0.76; data not shown). Total leukocyte (*P =* 0.90), monocyte (*P* = 0.87), and granulocyte (*P* = 0.84) numbers were unchanged after consumption of the L-SCFA and H-SCFA diets (**Supplemental Figure 5**). Although median (IQR) total lymphocyte numbers were not statistically different (*P* = 0.14) after the H-SCFA diet [1788 (519) cells/µL] than after the L-SCFA diet [1853 (863) cells/µL], they were investigated further with detailed analysis of B- and T-cell subsets.

**FIGURE 5:**
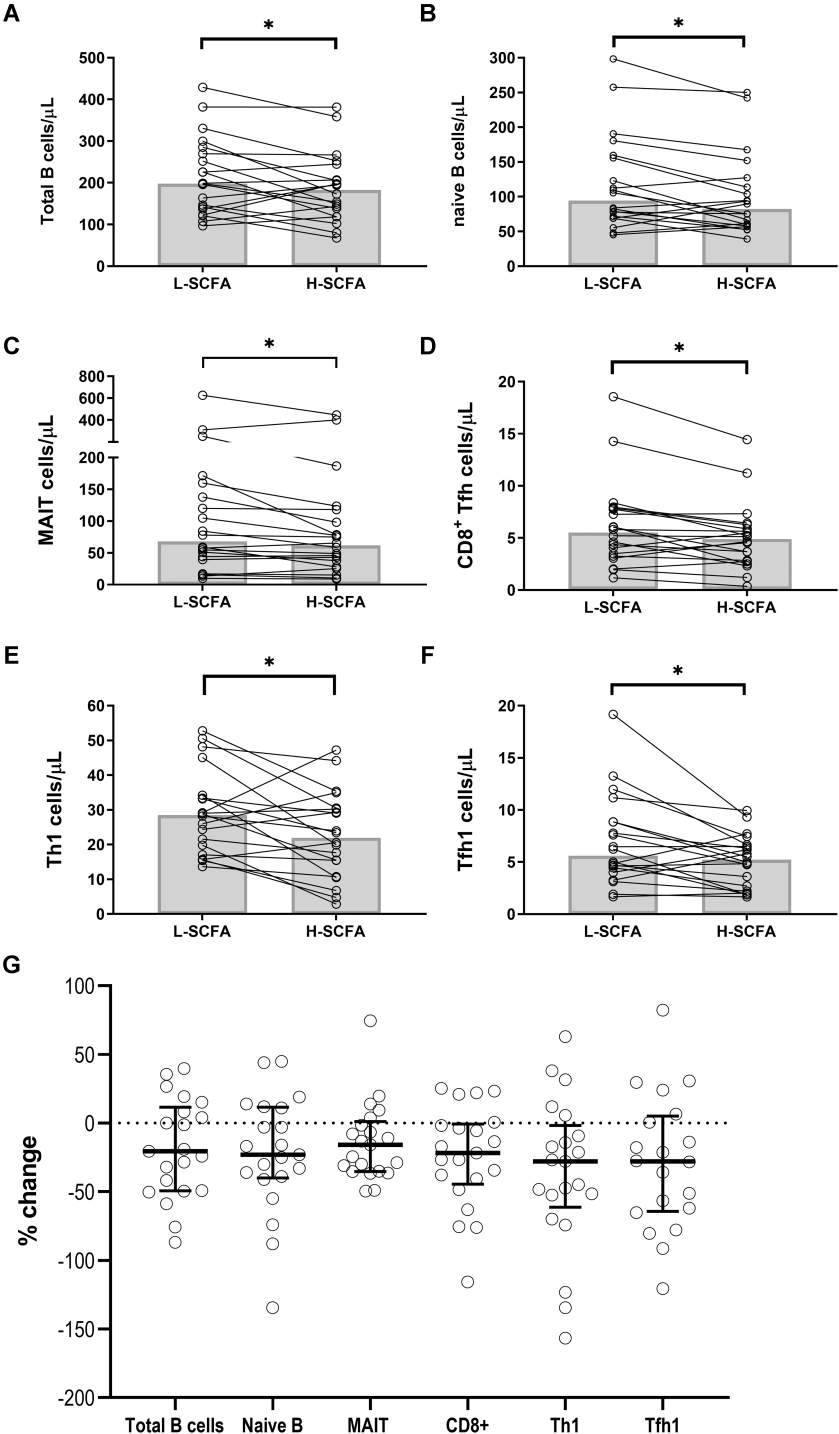
Significant changes to immune cell subsets associated with intervention diets. Paired changes to absolute numbers of (A) total B cells, (B) naive B cells, (C) MAITs, (D) CD8 + Tfh cells, (E) Th1 cells, and (F) Tfh1 cells as calculated from flow cytometry with BD Trucount tubes used to quantify absolute numbers. Bar represents median. (G) Relative changes to above subsets [(H-SCFA – L-SCFA)/H-SCFA]. *P = 0.05 (paired samples Wilcoxon test). *N* = 20 matched pairs. H-SCFA, high-SCFA; L-SCFA, low-SCFA; MAIT, mucosal-associated invariant T cell; Tfh, T follicular helper; Th, T helper.

**FIGURE 6:**
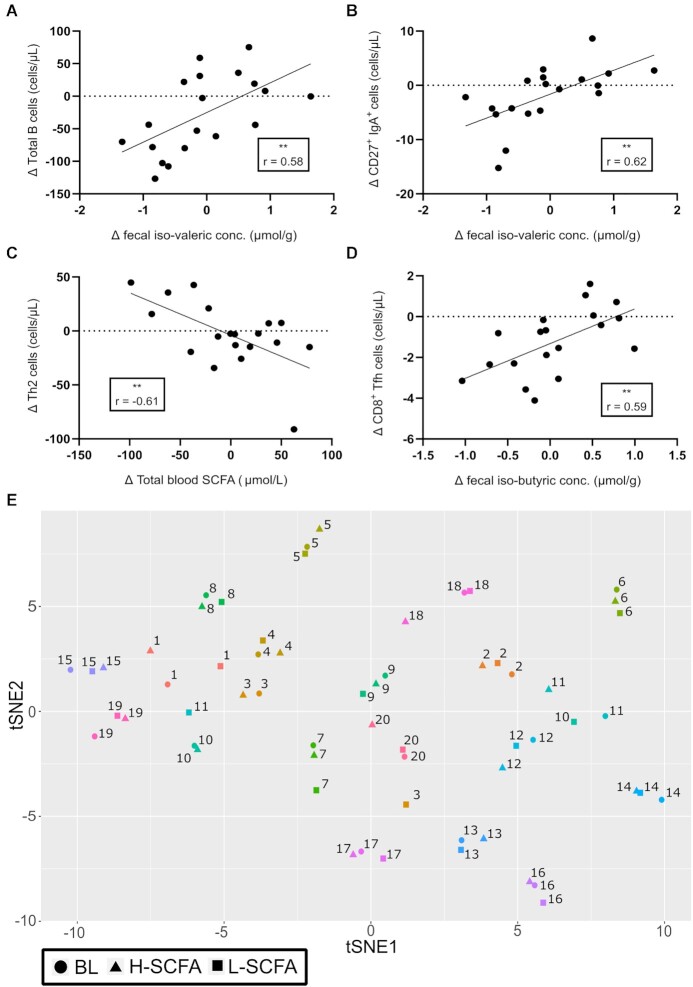
Correlations between individual immune cell subsets and global changes to overall immune profile. Correlations between SCFA and immune cell subsets (A–D). Δ, difference between H-SCFA and L-SCFA. Pearson correlation test: **P < 0.01 (*n* = 19). (E) t-SNE plot of global changes to immune parameters between BL and intervention diets. Numbers representative of each study participant (*N* = 20). BL, baseline; H-SCFA, high-SCFA; L-SCFA, low-SCFA; t-SNE, t-distributed stochastic neighbor embedding.

B cells and subsets therein were defined per **Supplemental Table 5** and gated using the strategy outlined in **Supplemental Figure 6** (20). Total B-cell numbers were lower after the H-SCFA diet than after the L-SCFA diet [median (IQR), 184 (112) compared with 199 (143) cells/µL, *P* < 0.05; **Figure 5**A]. This was equivalent to a median relative change of –20.6% (60.9%) from the L-SCFA to H-SCFA diet (Figure 5G). The difference (∆) in B-cell number between the H-SCFA and L-SCFA diets was positively correlated with the difference in fecal isovaleric concentrations (*P* < 0.01, *q* = 0.1, *r* = 0.58; 95% CI: 0.18, 0.82; **Figure 6**A). Detailed B-cell subsetting (**Supplemental Figure 7**) showed lower naive mature B-cell numbers after the H-SCFA diet than after the L-SCFA diet [82.5 (65.8) compared with 94.7 (88.9) cells/µL, *P* < 0.05; Figure 5B], whereas absolute numbers of transitional B cells, memory B-cell subsets, plasma cells, and CD21^lo^ B cells were not different between the intervention diets. However, positive correlations were found between changes in numbers of plasma cells (*P* < 0.01, *q* = 0.08, *r* = 0.59; 95% CI: 0.19, 0.83) and transitional B cells (*P* < 0.01, *q* = 0.049, *r* = 0.65; 95% CI: 0.27, 0.85) with changes in fecal SCFA concentrations between the H-SCFA and L-SCFA diets (data not shown). Furthermore, changes to the memory B-cell subsets—CD27^+^IgA^+^ (*P* < 0.01, *q* = 0.07, *r* = 0.62; 95% CI: 0.23, 0.84; Figure 6B) and CD27^+^IgG^+^ (*P* < 0.01, *q* = 0.07, *r* = 0.62; 95% CI: 0.22, 0.84; data not shown)—positively correlated with changes to fecal isovaleric concentrations between the intervention diets.

T-cell subsets were defined as before (20), listed in **Supplemental Table 6**, and gated according to Supplemental Figure 7. Total T-cell numbers were not statistically different after the H-SCFA diet than after the L-SCFA diet [median (IQR), 1233 (543) compared with 1253 (619) cells/µL; *P* = 0.12; Supplemental Figure 4], although 12 of 20 participants had lower T-cell numbers. This was examined further with detailed analysis of T-cell subsets (**Supplemental Figure 8**). Significantly lower numbers of the following were found after the H-SCFA diet as compared with the L-SCFA diet: mucosal-associated invariant T cells [MAITs; median (IQR), 62 (83) compared with 69 (114) cells/µL], CD8^+^ T follicular helper (Tfh) cells [4.9 (3.4) compared with 5.5 (4.5) cells/µL], T helper 1 (Th1) cells [22 (19) compared with 29 (16) cells/µL], and Tfh1 cells [5.2 (4.3) compared with 5.6 (4.7) cells/µL; Figure 5C–F]. Changes to Th2 cell numbers between the H-SCFA and L-SCFA diets (Figure 6C) were negatively correlated (*P* < 0.01, *q* = 0.08, *r* = –0.61; 95% CI: –0.83, –0.22) to changes in blood total SCFA concentrations. Furthermore, changes to CD8^+^ Tfh cells were positively correlated (*P* < 0.01, *q* = 0.09, *r* = 0.59; 95% CI: 0.18, 0.82) with changes in fecal isobutyric concentration (Figure 6D).

Overall changes to the phenotype of the immune system were visualized by plotting all immune cell subsets with a t-distributed stochastic neighbor embedding approach. Changes to the overall immune phenotype were found to cluster to each individual, as opposed to clustering by intervention diet (Figure 6E).

## Discussion

We here demonstrated with a controlled dietary intervention that high dietary intake of SCFAs and fibers can increase total fecal SCFA concentrations and reduce fecal ammonia, consistent with an increase in and reduction of the colonic fermentation of fiber and protein, respectively. Furthermore, increases in plasma propionate and butyrate were observed after the H-SCFA diet, whereas acetate and total SCFA concentrations were unchanged. The H-SCFA diet was associated with changes in the circulating B- and T-cell compartments, with significant reductions in numbers of B cells, Th1 cells, Tfh1 cells, and MAITs. However, the magnitudes of these differences were small, and overall modulation of immune parameters tended to be unique to each individual.

Building on our previous work (12), we hypothesized that dietary fiber and oral SCFA supplementation would increase delivery of SCFA to the systemic circulation. Indeed, colonic fermentation of carbohydrates was highly likely to be increased in the H-SCFA relative to the L-SCFA dietary period. First, the delivery of fiber to the colon was successful, as reflected in *1*) good compliance to the diets; *2*) the greater colonic gas production experienced by the participants, as anticipated from other studies using, for example, RS and inulin (22, 23); and *3*) the higher fecal output and proportion of stools that could be classified within 3–5 on the BSS. Second, fecal acetate and butyrate concentrations were greater relative to those associated with the L-SCFA diet. Third, protein fermentation was suppressed, as shown by a higher SCFA:branched-chain fatty acid ratio and reduced ammonia concentrations (24). However, we did not achieve a significant increase to overall plasma SCFA concentrations. This most likely relates to the low bioavailability of SCFAs to the systemic circulation given that, following oral ingestion or production in the colonic lumen, the majority of SCFAs will be metabolized by colonocytes or hepatocytes on their first pass. Indeed, systemic availability of colonic acetate, propionate, and butyrate has been estimated to be 36%, 9%, and 2%, respectively (25). After consumption of a vinegar drink containing 1.5 g of acetate, plasma acetate concentrations may transiently increase within 60 min, indicative of rapid absorption in the upper gastrointestinal tract (2). In contrast, colonic fermentation of inulin, primarily in the cecum, produces a plasma SCFA peak approximately 4–6 h after consumption (13). Consumption of RS may also increase plasma SCFA for >6 h as it is slowly fermented, although previous studies have examined different forms of RS (e.g., pearl barley kernels, Nutriose) than the current study (13, 26). Such observations indicate that timing of blood sampling and participant meal consumption is critical if plasma SCFA concentrations are to be used as a marker of increased delivery of SCFA to the systemic circulation. These may not have been adequately controlled in this study to capture the transient increases to rapidly metabolized plasma SCFA (e.g., acetate) with a single measurement in all participants. The limited ability of plasma SCFA to reflect marked increases in delivery of SCFA during fermentable fiber supplementation also manifested in a feeding study of patients with irritable bowel syndrome in whom fecal, symptomatic, and local fermentation characteristics were evaluated per a novel telemetric gas-sensing capsule (27, 28). Furthermore, variation in fecal SCFA concentration may occur due to the heterogenous nature of the sample, which was overcome by pooling fecal samples, despite the caveat of requiring an additional freeze-thaw step during processing (29).

A novel aim of this dietary intervention study was to use detailed immunophenotyping to examine changes to the immune system that may have occurred as result of increased delivery of SCFA to the systemic circulation. We observed that naive B cells, Th1 cells, Tfh1 cells, and MAITs were significantly lower after the H-SCFA diet when compared with the L-SCFA diet. This may be suggestive of an anti-inflammatory effect of increased systemic SCFA delivery, in line with decreased peripheral blood inflammatory markers observed to occur in healthy people provided with prebiotic fiber supplements (30). Indeed, healthy people who followed a high-fiber diet for 14 wk had increased fecal butyrate concentrations that correlated with decreases to overall B-cell frequency, a similar result to what we have observed after 3 wk of controlled interventional diets (31). Increased naive B-cell numbers have been observed in the peripheral blood of children with inflammatory bowel disease (IBD), which may be due to impaired conversion to memory B cells (32, 33). Indeed, SCFAs are potent at inducing Ig class–switched B-cell subsets in animal models (34, 35). Although no changes to memory B-cell subsets were observed in our study, further intervention studies should examine whether increasing systemic SCFA delivery affects B-cell maturation during an immune challenge such as vaccination.

Observations from mouse models suggested that delivery of SCFA to the systemic circulation may induce protective Treg cells, particularly in the gut (36, 37). We did not observe any significant changes to subsets of Treg between the intervention diets. Indeed, SCFA may directly upregulate the Treg transcription factor FoxP3, which was not included in our phenotyping panel (38). However, we did observe significantly lower MAIT numbers, a recently identified T-cell subset associated with gut-mucosal tissue that responded to gut microbiota–derived vitamin B metabolites (39). These cells would be exposed to high concentrations of SCFA in this environment, although it is unclear if human MAITs directly respond to SCFA as other gut-associated subsets such as T-cell receptor ɣδ^+^ cells do (40). Modulation of MAIT numbers in the peripheral blood may have been induced by altered microbial composition and function in the gut resulting from the intervention diets. This may have led to migration of MAITs out of the peripheral blood to the gut, as has been shown to occur in response to enteric bacterial or viral infection (41). Similarly, Th1 cells were significantly lower after the H-SCFA diet than after the L-SCFA diet. A recent study that performed similar immunophenotyping in healthy people found reduced numbers of Th1 cells in the peripheral blood of individuals who had increased species of SCFA-producing bacteria *Ruminococcus*, which may be further indicative of an indirect effect of the intervention diets (42).

It is important to note that the magnitude of changes observed in this study was small, with network analysis via t-distributed stochastic neighbor embedding, highlighting that overall changes to the immune cell populations clustered within each individual. Furthermore, the small size of the study cohort limited the statistical power of changes observed. Up to 80% of immune variation in humans may be related to nonheritable lifestyle factors, which cannot be completely controlled through a 10-wk intervention study (43). Previous studies that have performed detailed immunophenotyping in patients with IBD or primary immune deficiency generally observed larger differences in T and B numbers (>20%) when compared with healthy controls (20, 33). Nevertheless, we did find that median relative changes in overall B cells, naive B cells, and some T-cell subsets between the diets were in the range of 15%–30% (Figure 5G). Increasing systemic delivery of SCFA through a dietary approach may have added benefit for those with IBD, as shifts in colonic fermentation observed through our dietary approach have been associated with anti-inflammatory effects in Crohn disease and ulcerative colitis (44). Our diet was well tolerated by the healthy cohort of study participants, although further studies will need to assess tolerability in patients with IBD, who may already have lower habitual dietary fiber intake than a healthy cohort and have reduced compliance to dietary intervention many weeks in duration (45, 46). A pilot study in small group of adults with multiple sclerosis found that 12 mo of following a high-fiber, vegetable-predominant diet significantly altered peripheral blood CD4^+^ T cells and improved clinical disease indices, highlighting that long-term dietary therapy to increase systemic SCFA may have more efficacy in other immune-mediated disorders (47).

In conclusion, dietary intervention with fermentable fiber and oral SCFA to increase colonic and peripheral blood SCFA is a well-tolerated intervention that is associated with favorable changes to colonic fermentation, reducing fecal ammonia. Increased SCFA was also associated with changes to the distribution of lymphocyte subsets, with lower naive B cells, MAITs, and Th1 cells in the peripheral blood. Further studies are needed in larger cohorts to assess if phenotypical changes are also associated with changes to immune function to determine if dietary therapy may have therapeutic benefit in patients with inflammatory disease.

## Supplementary Material

nqac246_Supplemental_File

## Data Availability

Data described in the article, codebook, and analytic code will be made available upon request to the corresponding author.

## Notes

PAG was supported by a scholarship from the Central Clinical School, Monash University; JGM by a Senior Research Fellowship (National Health and Medical Research Council; GNT1136988); and MCvZ by a Senior Research Fellowship (National Health and Medical Research Council; GNT1117687).

Author disclosures: PAG, JGM, and PRG work in a department that financially benefits from the sales of a digital application and booklets on the low FODMAP diet. They have published an educational/recipe book on diet. Funds raised contribute to research of the Department of Gastroenterology and to the university. The authors receive no personal remuneration. MCvZ reports no conflicts of interest.

Supplemental Tables 1–6 and Supplemental Figures 1–8 are available from the “Supplementary data” link in the online posting of the article and from the same link in the online table of contents at https://academic.oup.com/ajcn/.

Abbreviations used: BL, baseline; BSS, Bristol Stool Scale; H-SCFA, high-SCFA; IBD, inflammatory bowel disease; L-SCFA, low-SCFA; MAIT, mucosal-associated invariant T cell; PBMC, peripheral blood mononuclear cells; RS, resistant starch; Tfh, T follicular helper; Th, T helper; Treg, regulatory T cell.
