# Supplementary material for: A randomized dietary intervention to increase colonic and peripheral blood SCFAs modulates the blood B- and T-cell compartments in healthy humans
Source: Am J Clin Nutr. 2022 Sep 9;116(5):1354–67. doi: 10.1093/ajcn/nqac246 (PMC9630882; doi:10.1093/ajcn/nqac246)
Supplement: nqac246_Supplemental_File [file nqac246_supplemental_file.docx]

A randomised dietary intervention to increase colonic and peripheral blood short-chain fatty acids modulates the blood B- and T-cell compartments in healthy humans

Paul A. Gill

**Supplementary information**

**List of Tables**

| **Table** | **Page** |
| --- | --- |
| Supplementary Table 1. List of additional snacks recommended for consumption on intervention diet | 2 |
| Supplementary Table 2. Peripheral blood mononuclear cell staining panel | 3 |
| Supplementary Table 3. Daily Macronutrient and fibre intake during study intervention periods. | 4 |
| Supplementary Table 4. Faecal metabolite concentrations and daily output during intervention diets | 5 |
| Supplementary Table 5. Definition of B cell subsets | 6 |
| Supplementary Table 6. Definition of T cell subsets | 7 |

**List of Figures**

| **Figure** | **Page** |
| --- | --- |
| Supplementary Figure 1. Gating strategy to quantify leucocyte and lymphocyte populations in whole blood using Trucount beads | 8 |
| Supplementary Figure 2. Dietary compliance scores | 9 |
| Supplementary Figure 3. Gastrointestinal symptoms of study cohort during study | 10 |
| Supplementary Figure 4. Absolute numbers of T cell subsets from PBMC | 11 |
| Supplementary Figure 5. Absolute numbers are major leukocyte populations from whole blood after consumption of the low-SCFA (L-SCFA) and high-SCFA (H-SCFA) diets. | 12 |
| Supplementary Figure 6. Gating strategy to define B cell subsets | 13 |
| Supplementary Figure 7. Major B cell subset concentrations after each intervention diet | 15 |
| Supplementary Figure 8. Gating strategy to define T cell subsets | 16 |

## Supplementary Table 1. List of additional snacks recommended for consumption on intervention diet

| **Snack** | **Recommended** |
| --- | --- |
| Biscuits, crackers, bread | plain sweet biscuits, savoury crackers (Savoys, rice crackers, water crackers), white bread, crumpets, scones |
| Meat & Poultry | chicken, beef, pork, ham, bacon, eggs |
| Dairy | Yoghurt, cheese, milk, chocolate |
| Spreads | Peanut butter, vegemite, strawberry jam, honey |
| Drinks: | orange juice, milk, tea, coffee, water, alcohol (not to excess) |
| Other | Please discuss with your researcher |

## Supplementary Table 2. Peripheral blood mononuclear cell staining panel

| **Channel** | **B cells** | | | **T cells** | | |
| --- | --- | --- | --- | --- | --- | --- |
|  | **Marker (*clone*)** | **Source** | **Volume in test (µL)** | **Marker *(clone)*** | **Source** | **Volume in test (µL)** |
| **BUV395** | - | - | - | CXCR3  *(1C6)* | BD Biosciences | 3 |
| **BUV496** | - | - | - | CD4  *(SK3)* | BD Biosciences | 1.5 |
| **BUV737** | - | - | - | CD45RA  *(HI100)* | BD Biosciences | 0.5 |
| **BV421** | CD27  (*M-T271*) | BD Biosciences | 1 | CD25  *(BC96)* | Biolegend | 2.5 |
| **BV510** | IgM  *(MHM-88)* | Biolegend | 1 | CD161  *(HP-3G10)* | Biolegend | 2.5 |
| **BV605** | *-* | - | - | Vα7.2  *(3C10)* | BD Biosciences | 1 |
| **BV711** | CD21  *(B-ly4)* | BD Biosciences | 5 | CD3  *(UCHT1)* | BD Biosciences | 2.5 |
| **BV785** | IgG  *(G18-145)* | BD Biosciences | 1 | TCRγδ  *(11F2)* | BD Biosciences | 2.5 |
| **FITC** | IgA  *(IS11-8E10)* | Miltenyi | 0.5 | CXCR5  *(RF8B2)* | BD Biosciences | 5 |
| **PerCP-Cy5.5** | IgD  *(IA6-2)* | Biolegend | 1.5 | CCR6  *(G034E3)* | Biolegend | 2.5 |
| **PE** |  |  |  |  |  |  |
| **PE-CF594** |  |  |  | CD127  *(HIL-7R-M21)* | BD Biosciences | 5 |
| **PE-Cy7** | CD19  *(SJ25C1)* | BD Biosciences | 5 | CCR4  *(L291H4)* | Biolegend | 5 |
| **APC** |  |  |  |  |  |  |
| **AF-700** | - | - | - | - | - | - |
| **APC-H7** | CD38  *(HB-7)* | Biolegend | 0.2 | CD8  *(SK1)* | BD Biosciences | 5 |
| *BUV: Blue-ultra violet, BV: blue-violet, CCR: C-C chemokine receptor, TACI: Transmembrane activator and calcium-modulating cyclophilin ligand interactor, ICOS: Inducible T-cell co-stimulator, TCR: T-cell receptor, HLA: Human leucocyte antigen.* | | | | | | |

## Supplementary Table 3. Daily Macronutrient and fibre intake during study intervention periods.

| **Daily intake** | **Low-SCFA** | **High-SCFA** |
| --- | --- | --- |
| Energy (kj) | 9101 (4739-10311) | 8263 (5503-11485) |
| Carbohydrate (g) | 233.0 (127.0-266.1) | 215.1 (141.3-296.4) |
| - *Sugars (g)* | 76.0 (37.6-102.2) | 72.2 (49.3-119.6) |
| Protein (g) | 99.8 (60.4-121.5) | 93.8 (65.3-127.2) |
| Fat (g) | 88.2 (40.9-99.3) | 83.6 (52.5-107.5) |
| - *Saturated Fat (g)* | 31.7 (12.8-40.6) | 30.5 (19.0-43.9) |
| Sodium (mg) | 3081 (1859-4025) | 3177 (1913-4004) |
| Total dietary fibre (g)   - *Oligosaccharide (g)* - *Resistant starch (g)* - *Inulin (g)* | 18.7 (10.1-23.6)  1.2 (0.6-1.8)  1.0 (0.6-1.3)  0 | **36.5 (22.3-44.9)******  1.0 (0.6-2.2)  **10.5 (5.0-11.6)******  **7.4 (5.3-8.1)****** |
| Proportion of total energy intake (%) |  |  |
| - *Fat* | 35.6 (31.9-37.5) | 35.8 (33.3-37.9) |
| - *Carbohydrate* | 43.9 (41.3-47.8) | 43.3 (40.8-45.6)** |
| - *Protein* | 19.5 (16.3-21.7) | 19.0 (15.9-20.2)* |
| **Total dietary fibre includes RS, inulin and oligosaccharides. Data shown as median (range), statistical significance compared to low-SCFA calculated by Wilcoxon test denoted by *P<0.05, **P<0.01, ****P<0.001. n=20.* | | |

| **Metabolite** | **Concentration (µmol/g)** | |  | **Amount (mmol/day)** | |  |
| --- | --- | --- | --- | --- | --- | --- |
|  | Low-SCFA | High-SCFA | *P* | Low-SCFA | High-SCFA | *P* |
| Total SCFA   - Acetate - Propionate - Butyrate - Valerate - Caproate | 75.3 (37.6-156.6)  41.4 (19.5-96.9)  17.1 (4.7-25.7)  11.9 (5.0-31.9)  1.7 (0.1-2.9)  0.3 (0.0-3.5) | 86.6 (37.7-153.8)  48.8 (21.4-104.6)  16.3 (8.2-35.1)  16.6 (4.9-25.0)  1.9 (0.1-3.6)  0.5 (0.0-2.7) | **0.02**  **0.03**  0.35  0.07  0.47  0.27 | 6.5 (1.4-31.3)  3.8 (0.9-20.1)  1.6 (0.2-5.7)  1.0 (0.2-4.9)  0.2 (0.1-0.6)  0.03 (0.00-0.30) | 8.7 (1.5-29.1)  5.0 (0.8-17.5)  2.0 (0.3-5.2)  1.5 (0.4-6.2)  0.3 (0.1-0.7)  0.04 (0.00-0.56) | 0.19  0.18  0.46  0.25  0.47  0.24 |
| Total BCFA   - Iso-butyrate - Iso-valerate | 4.1 (2.4-7.2)  1.6 (1.1-2.8)  2.5 (1.3-4.7) | 4.2 (2.0-9.7)  1.7 (0.9-3.6)  2.4 (1.1-6.0) | 0.86  0.68  0.65 | 0.4 (0.1-0.9)  0.2 (0.1-0.3)  0.2 (0.1-0.6) | 0.5 (0.1-1.1)  0.2 (0.1-0.5)  0.3 (0.1-0.7) | 0.44  0.29  0.47 |
| Ammonia | 33.4 (16.7-52.8) | 26.2 (11.8-55.2) | **0.04** | 2.4 (1.0-6.6) | 2.8 (1.0-7.5) | 0.44 |
| *BCFA: Branched-chain fatty acid. SCFA, BCFA and ammonia amount as determined by concentrations normalised to daily faecal output. Statistical significance as calculated by Wilcoxon test, bolded P-values statistically significant. Data shown as median±range. N=19* | | | | | | |

## Supplementary Table 4. Faecal metabolite concentrations and daily output during intervention diets

*.*

## Supplementary Table 5. Definition of B cell subsets

| **Population name** | **Phenotype definition** |
| --- | --- |
| B cells | FSC^low^SSC^low^CD19^+^ |
| transitional | FSC^low^SSC^low^CD19^+^CD38^hi^CD27^-^ |
| naive mature | FSC^low^SSC^low^CD19^+^CD38^lo^CD21^+^CD27^-^IgM^+^IgD^+/-^ |
| Natural effector | FSC^low^SSC^low^CD19^+^CD38^lo^CD21^+^CD27^+^IgM^+^IgD^+^ |
| Memory B cells   - Natural effector - CD27^+^ IgM^+^ - CD27^+^ IgG^+^ - CD27^+^ IgA^+^ - CD27^-^ IgG^+^ - CD27^-^ IgA^+^ | FSC^low^SSC^low^CD19^+^CD38^lo^CD21^+^CD27^+^IgM^+^IgD^+^  FSC^low^SSC^low^CD19^+^CD38^lo^CD21^+^CD27^+^IgM^+^IgD^-^  FSC^low^SSC^low^CD19^+^CD38^lo^CD21^+^CD27^+^IgM^-^IgD^-^IgG^+^  FSC^low^SSC^low^CD19^+^CD38^lo^CD21^+^CD27^+^IgM^-^IgD^-^IgA^+^  FSC^low^SSC^low^CD19^+^CD38^lo^CD21^+^CD27^-^IgM^-^IgD^-^IgG^+^  FSC^low^SSC^low^CD19^+^CD38^lo^CD21^+^CD27^-^IgM^-^IgD^-^IgA^+^ |
| plasma cells | FSC^low^SSC^low^CD19^+^CD38^hi^CD27^+^ |
| CD21^low^ B cells | FSC^low^ SSC^low^ CD19^+^CD38^lo^CD27^-^CD21^low^ |

## Supplementary Table 6. Definition of T cell subsets

| **Population name** | **Phenotype definition** |
| --- | --- |
| T cells | FSC^low^SSC^low^CD3^+^ |
| γδ^+^ T cells | FSC^low^SSC^low^CD3^+^γδ^+^ |
| MAIT cells | FSC^low^SSC^low^CD3^+^γδ^-^CD161^+^Vα7.2^+^ |
| CD8^+^ T cells | FSC^low^SSC^low^CD3^+^γδ^-^CD161^-^Vα7.2^-^CD8^+^ |
| - CD8 Tfh | FSC^low^SSC^low^CD3^+^γδ^-^CD161^-^Vα7.2^-^CD8^+^CXCR5^+^CD45RA^-^ |
| - CD8 memory | FSC^low^SSC^low^CD3^+^γδ^-^CD161^-^Vα7.2^-^CD8^+^CXCR5^-^CD45RA^-^ |
| - CD8 naïve | FSC^low^SSC^low^CD3^+^γδ^-^CD161^-^Vα7.2^-^CD8^+^CXCR5^-^CD45RA^+^ |
| CD4^+^ T cells | FSC^low^SSC^low^CD3^+^γδ^-^CD161^-^Vα7.2^-^CD4^+^ |
| - Treg cells | FSC^low^SSC^low^CD3^+^γδ^-^CD161^-^Vα7.2^-^CD4^+^CD25^+^CD127^lo/-^ |
| - - memory Treg | FSC^low^SSC^low^CD3^+^γδ^-^CD161^-^Vα7.2^-^CD4^+^CD25^+^CD127^lo/-^CCR4^+^CD45RA^-^ |
| - - naive Treg | FSC^low^SSC^low^CD3^+^γδ^-^CD161^-^Vα7.2^-^CD4^+^CD25^+^CD127^lo/-^CCR4^-^CD45RA^+^ |
| - - CD4 Tfr | FSC^low^SSC^low^CD3^+^γδ^-^CD161^-^Vα7.2^-^CD4^+^CD25^+^CD127^lo/-^CXCR5^+^CD45RA^-^ |
| - Th cells | FSC^low^SSC^low^CD3^+^γδ^-^CD161^-^Vα7.2^-^CD4^+^CD25^-^CD127^lo/hi^CXCR5^-^CD45RA^-^ |
| - - Th1   - Th2   - Th17   - Th22 | FSC^low^SSC^low^CD3^+^γδ^-^CD161^-^Vα7.2^-^CD4^+^CD25^-^CD127^lo/hi^CXCR5^-^CD45RA^-^CCR6^-^CXCR3^+^CCR4^-^  FSC^low^SSC^low^CD3^+^γδ^-^CD161^-^Vα7.2^-^CD4^+^CD25^-^CD127^lo/hi^CXCR5^-^CD45RA^-^CCR6^-^CXCR3^-^CCR4^+^  FSC^low^SSC^low^CD3^+^γδ^-^CD161^-^Vα7.2^-^CD4^+^CD25^-^CD127^lo/hi^CXCR5^-^CD45RA^-^CCR6^+^CXCR3^-^CCR4^+^  FSC^low^SSC^low^CD3^+^γδ^-^CD161^-^Vα7.2^-^CD4^+^CD25^-^CD127^lo/hi^CXCR5^-^CD45RA^-^CCR6^+^CXCR3^+^CCR4^-^ |
| - Tfh cells - Tfh1 - Tfh2 - Tfh17 - Tfh22 | FSC^low^SSC^low^CD3^+^γδ^-^CD161^-^Vα7.2^-^CD4^+^CD25^-^CD127^lo/hi^CXCR5^+^CD45RA^-^  FSC^low^SSC^low^CD3^+^γδ^-^CD161^-^Vα7.2^-^CD4^+^CD25^-^CD127^lo/hi^CXCR5^+^CD45RA^-^CCR6^-^CXCR3^+^CCR4^-^  FSC^low^SSC^low^CD3^+^γδ^-^CD161^-^Vα7.2^-^CD4^+^CD25^-^CD127^lo/hi^CXCR5^+^CD45RA^-^CCR6^-^CXCR3^-^CCR4^+^  FSC^low^ SSC^low^ CD3^+^γδ^-^CD161^-^Vα7.2^-^CD4^+^CD25^-^CD127^lo/hi^CXCR5^+^CD45RA^-^CCR6^+^CXCR3^-^CCR4^+^  FSC^low^ SSC^low^ CD3^+^γδ^-^CD161^-^Vα7.2^-^CD4^+^CD25^-^CD127^lo/hi^CXCR5^+^CD45RA^-^CCR6^+^CXCR3^+^CCR4^-^ |
| *MAIT: Muscosal-associated T cell* | |

**Supplementary Figures**

### Supplementary Figure 1. Gating strategy to quantify leucocyte and lymphocyte populations in whole blood using Trucount beads

CD45^+^ cells were differentiated based on side scatter area (SSC-A) into granulocytes (SSCA^hi^), monocytes (SSCA^med^) and lymphocytes (SSCA^lo^). Lymphocytes were further differentiated into natural killer (NK) cells (CD16^+^CD56^+^), B cells (CD19^+^) and T cells (CD3^+^). T cells were then assessed for CD4 and CD8 expression. Beads used for enumeration of these subsets were identified based on positivity for CD16,CD56 and CD3.


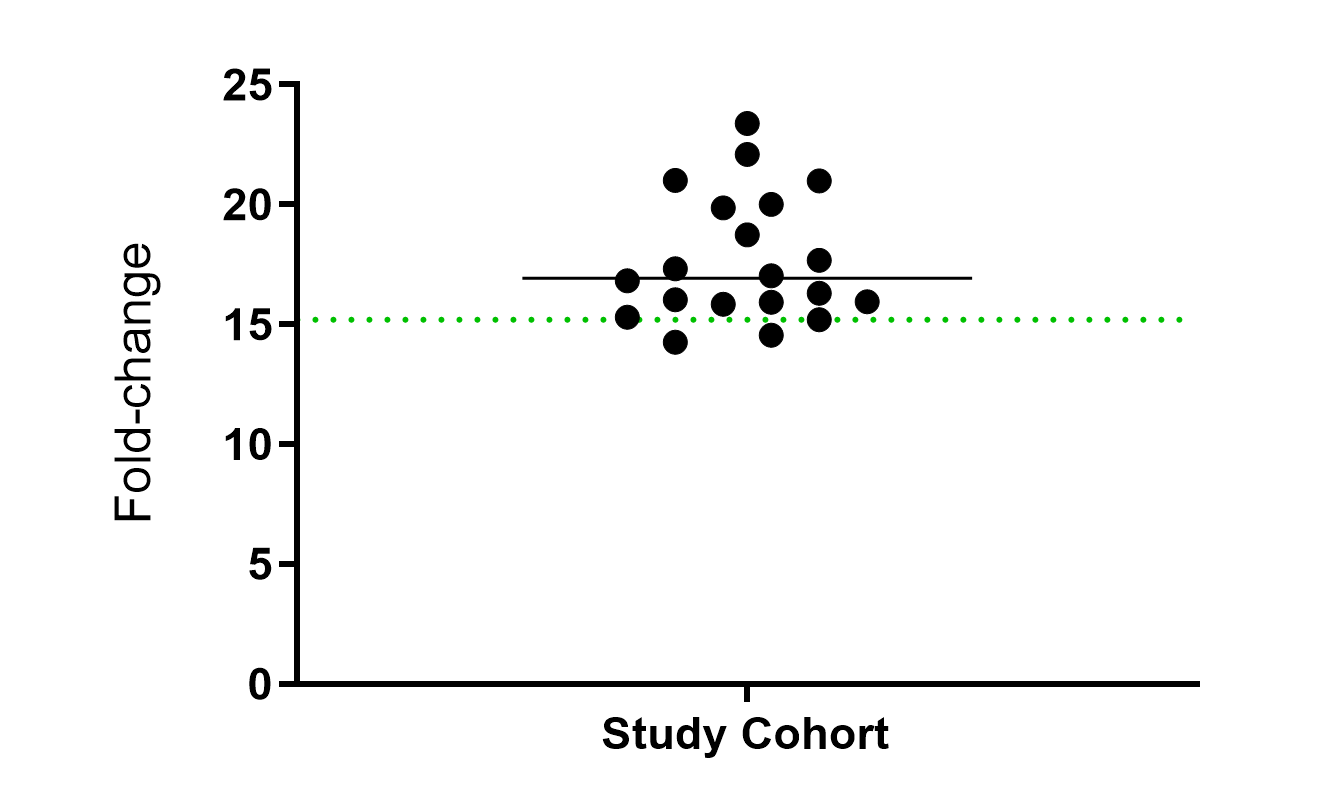


### Supplementary Figure 2. Dietary compliance scores

Compliance score calculated as fold-change in resistant starch and inulin intake from low-SCFA (L-SCFA) to high-SCFA (H-SCFA) diet. Green dotted line indicates the minimum 80% (x15.2 fold-change) cut-off defined for compliance. Data shown as median. *n*=20


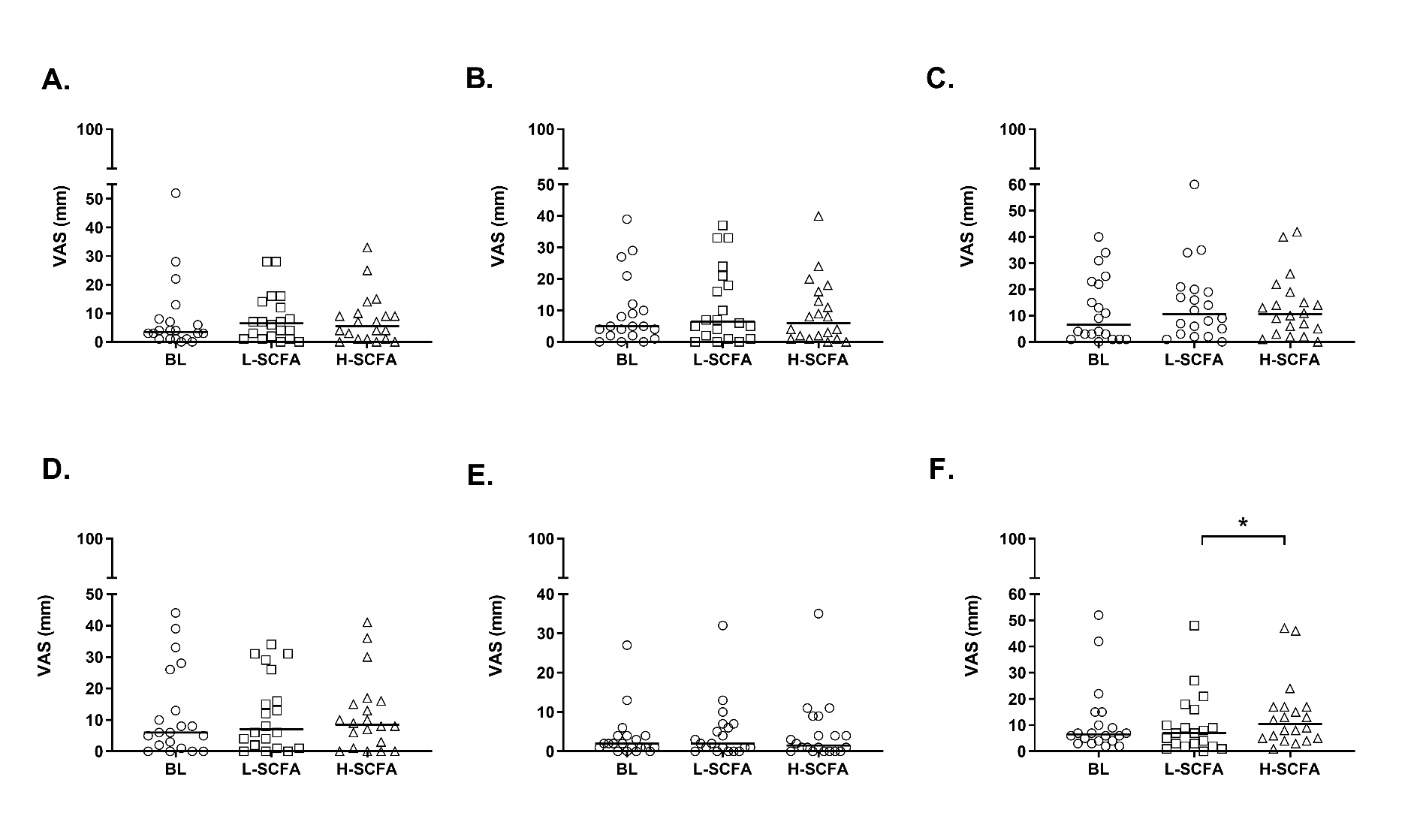


**Supplementary Figure 3.** Gastrointestinal symptoms of study cohort during study

Mean symptom scores of A) abdominal pain, B) abdominal bloating, C) faecal satisfaction, D) tiredness and lethargy, E) nausea and F) passage of wind derived from 100 mm visual-analogue scale (VAS) recorded for 21-days for low-SCFA (L-SCFA) and high-SCFA (H-SCFA) diet. Baseline (BL) data collected over 7 days. Data shown as median. Statistical significance as determined by Friedman test with Dunn’s multiple comparisons test denoted as **P*<0.05. *n*=20.


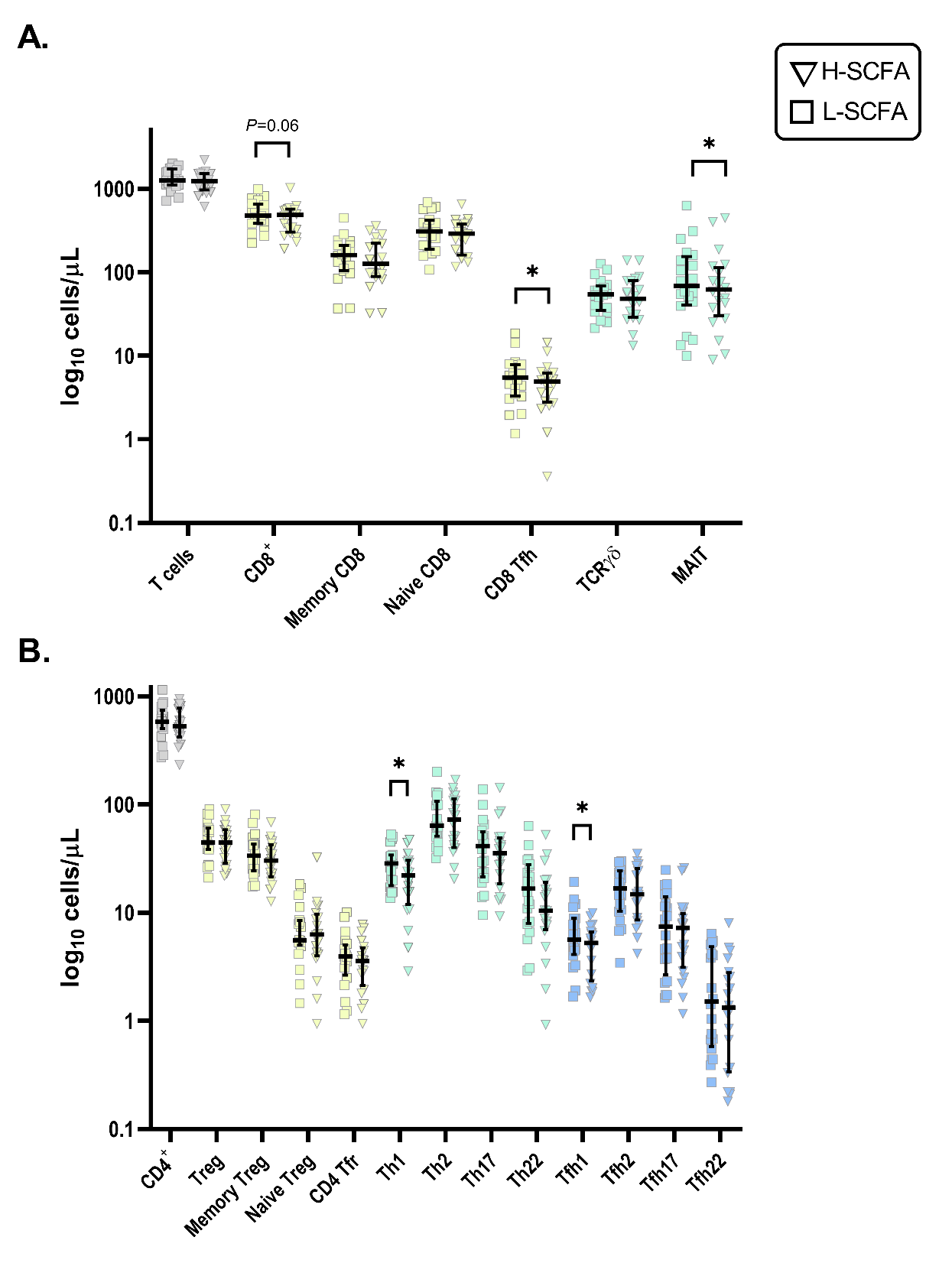


**Supplementary Figure 4.** Absolute numbers of T cell subsets from PBMC as calculated from flow cytometry with BD TruCount® tubes used to quantify T overall cells. **A)** Major T cell subsets after each intervention diet, grey: overall T cells, yellow: CD8^+^ T cells, green: gut-associated T cell subsets. **B)** T-helper subsets after each intervention diet. Grey: CD4^+^ cells, yellow: T-regulatory cell subsets, green: T-helper cell subsets, blue: T-follicular helper cell subsets. Th: T-helper cell, Treg: T-regulatory cell, TCR: T-cell receptor, MAIT: Mucosal-associated invariant T cell, L-SCFA: low-SCFA diet, H-SCFA: high-SCFA diet. Statistical significance as determined by Wilcoxon test. Data shown as median±IQR. **P*=0.05, n=20 matched pairs.


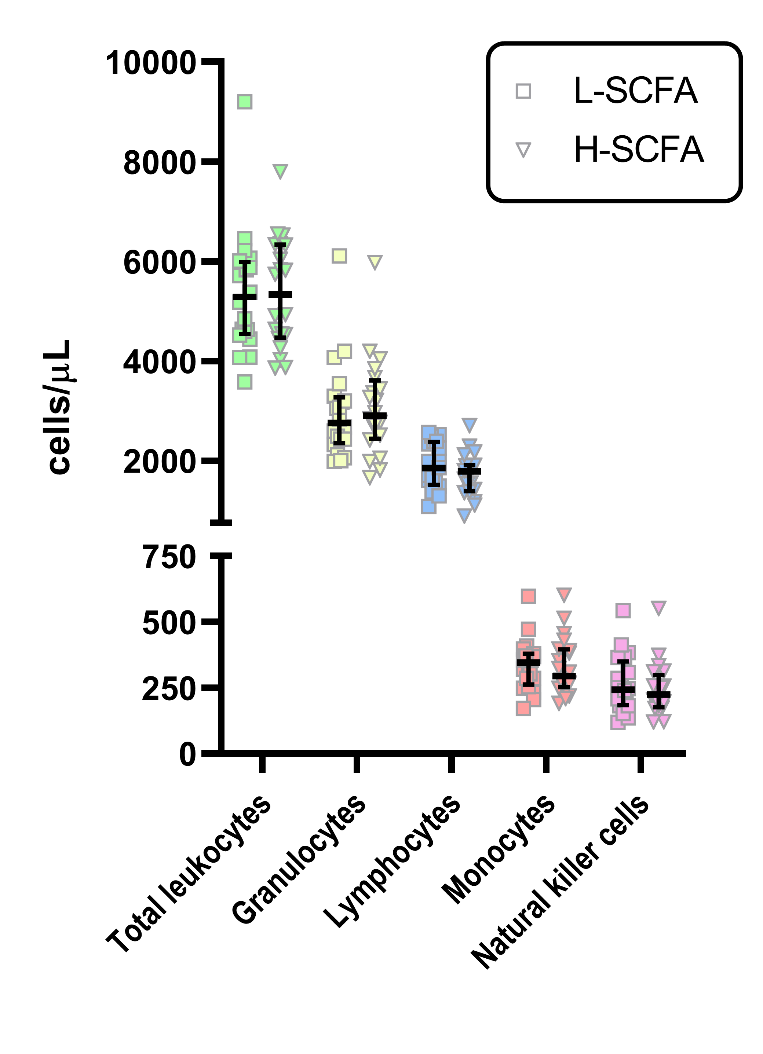


**Supplementary Figure 5.** Absolute numbers are major leukocyte populations from whole blood after consumption of the low-SCFA (L-SCFA) and high-SCFA (H-SCFA) diets. Absolute numbers of leucocyte subsets as calculated using BD Trucount® beads. No statistical significance as calculated by Wilcoxon test (n=20 matched pairs). Data shown as median±IQR.


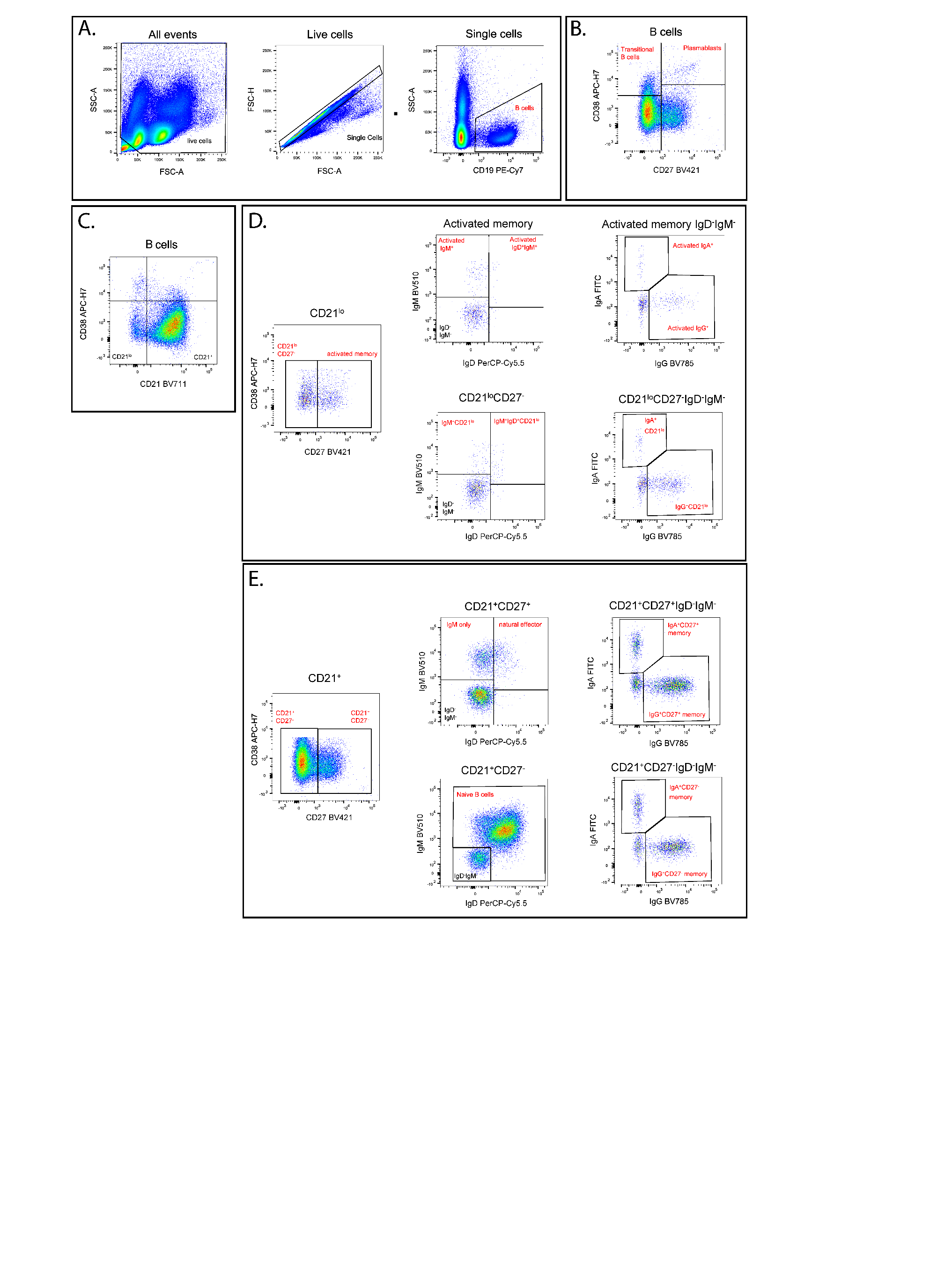


**Supplementary Figure 6.** Gating strategy to define B cell subsets

**A)** Gating strategy to define total CD19+ B cells. **B)** Transitional B cells and plasmablasts are delineated from B cells. **C)** In parallel, B cells are gated based on dim expression of CD38 and divided into CD21lo and CD21+ B cells. **D)** CD21lo B cells and **E)** CD21+ B cells are subdivided into CD27+ and CD27- and assessed based on IgD and IgM expression to delineate IgM only and IgM+IgD+ cells. The IgD-IgM- cells are further characterised according to IgA and IgG expression.


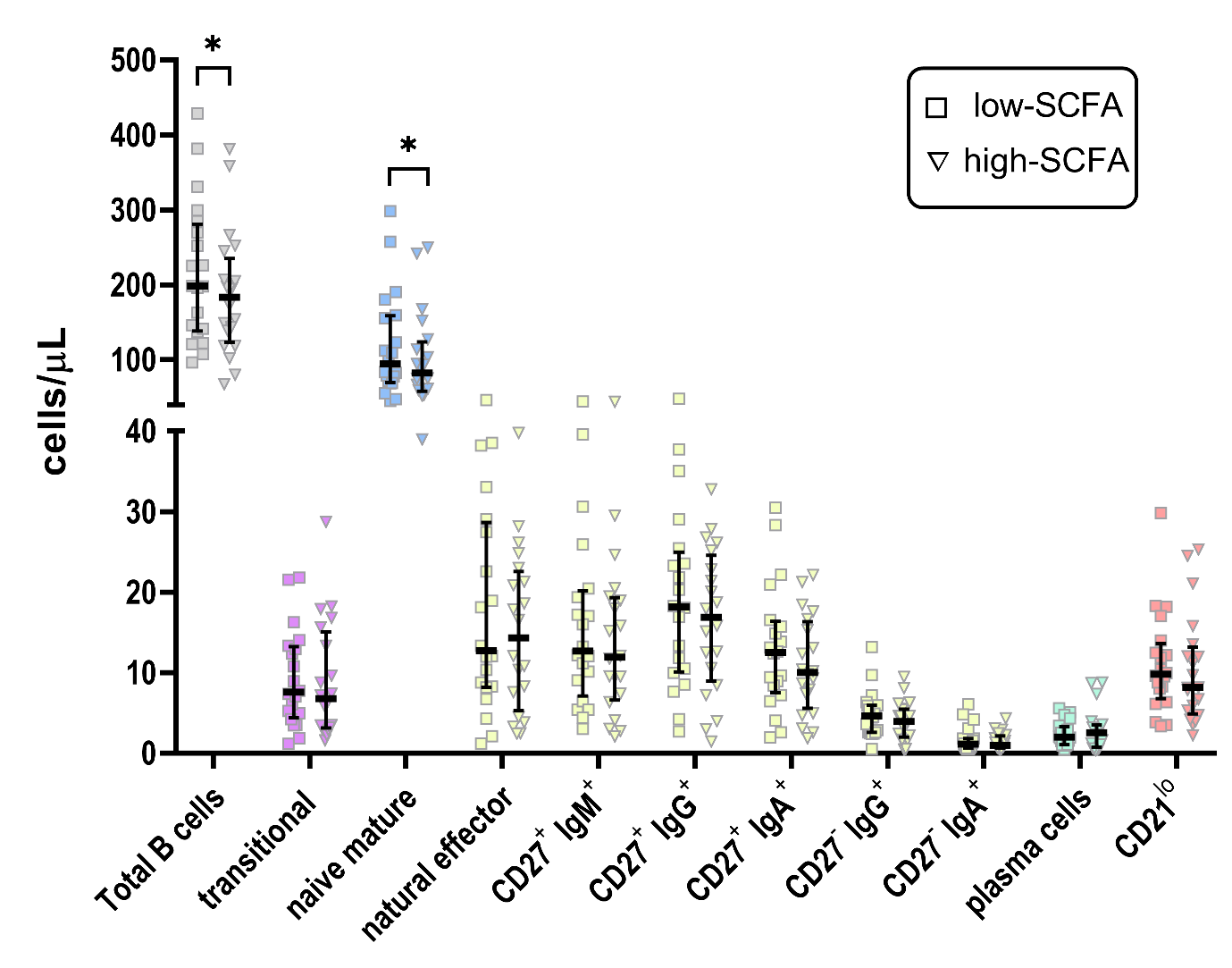


**Supplementary Figure 7.** Major B cell subset concentrations after each intervention diet

Absolute numbers of B cell subsets from PBMC as calculated from flow cytometry with BD TruCount® tubes used to quantify overall B cells. Grey: overall B cells, purple: transitional B cells, blue: naive mature cells, yellow: memory subsets, green: plasma cells, red: CD21^lo^. Statistical significance as determined by Wilcoxon test. Data shown as median±IQR. **P*=0.05, n=20 matched pairs.


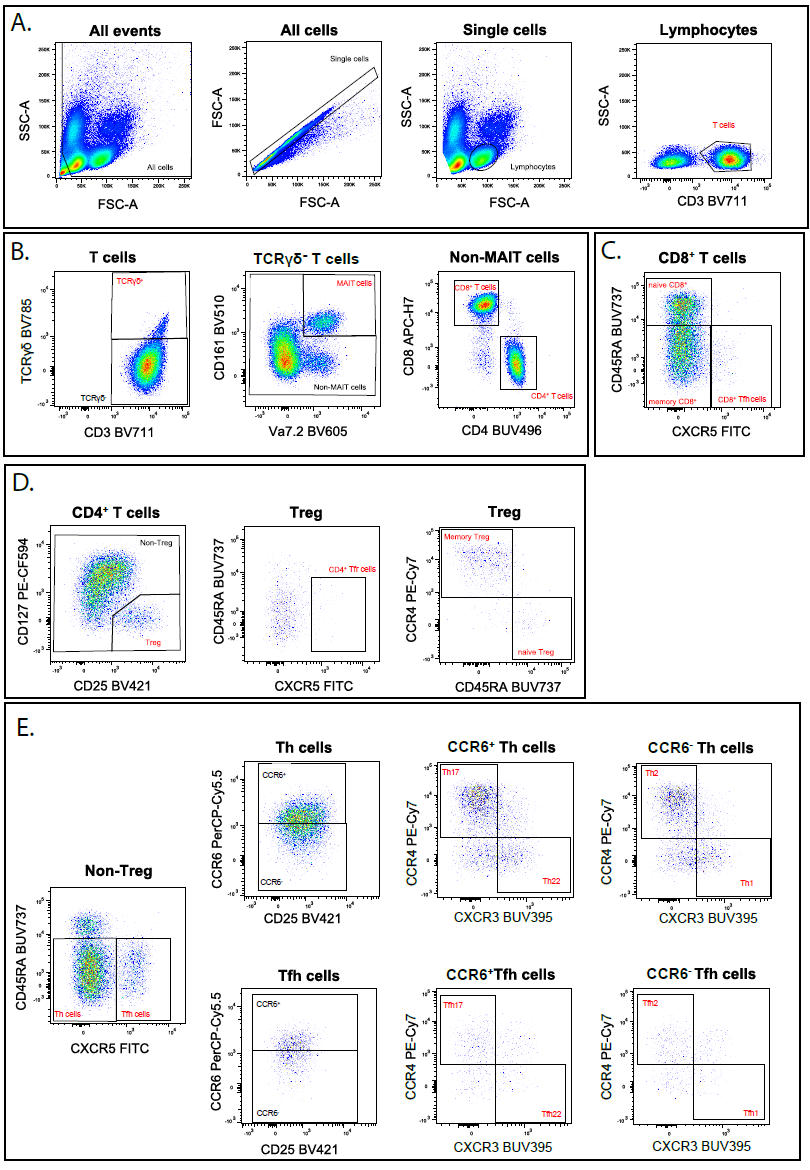


**Supplementary Figure 8.** Gating strategy to define T cell subsets

**A)** Gating strategy to define total CD3+ T cells. **B)** T cells are further gated to delineate TCRɣδ expressing cells, with the negative population assessed for CD161 and Vα7.2 expression to isolate mucosal-associated T (MAIT) cells. Non-MAIT cells are assessed for CD4 and CD8 expression. **C)** CD8+ T cell are examined for CD45RA and CXCR5 to identify naïve, memory and CD8 T-follicular helper (Tfh) subsets. **D)** CD4+ T cells are separated into Treg and non-Treg cells based on the expression of CD25 and CD127. Treg cells are then delineated into naïve, memory and T-follicular helper (Tfh) subsets**. E)** Non-Treg CD4+ T cells are separated into T-helper and T-follicular helper subsets, prior to parallel assessment for CCR6 expression. CCR6+ and CCR6- subsets from both Th and Tfh cells are then assessed for CXCR3 and CCR4 expression to delineate Th1, Th1, Th17 and Th22 subsets (and the respective Tfh subsets
